# Characterization of the microenvironment in different immune-metabolism subtypes of cervical cancer with prognostic significance

**DOI:** 10.3389/fgene.2023.1067666

**Published:** 2023-02-03

**Authors:** Wujiang Lai, Jinrong Liao, Xiaoxuan Li, Peili Liang, Liqing He, Keke Huang, Xiaomei Liang, Yifeng Wang

**Affiliations:** ^1^ Obstetrics and Gynecology Center, Zhujiang Hospital, Southern Medical University, Guangzhou, China; ^2^ Department of Obstetrics and Gynecology, Guangdong Provincial Key Laboratory of Major Obstetric Diseases, The Third Affiliated Hospital of Guangzhou Medical University, Guangzhou, China; ^3^ Center for Reproductive Medicine/Department of Fetal Medicine and Prenatal Diagnosis/BioResource Research Center, Guangdong Provincial Key Laboratory of Major Obstetric Diseases, The Third Affiliated Hospital of Guangzhou Medical University, Guangzhou, China; ^4^ Department of Obstetrics, Shunde Hospital, The First People’s Hospital of Shunde, Southern Medical University, Foshan, Guangdong, China

**Keywords:** cervical cancer, molecular subclassification, immune microenvironment, metabolic reprogramming, prognosis

## Abstract

**Introduction:** Immune cell infiltration and metabolic reprogramming may have great impact on the tumorigenesis and progression of malignancies. The interaction between these two factors in cervical cancer remains to be clarified. Here we constructed a gene set containing immune and metabolism related genes and we applied this gene set to molecular subtyping of cervical cancer.

**Methods:** Bulk sequencing and single-cell sequencing data were downloaded from the Cancer Genome Atlas (TCGA) database and Gene Expression Omnibus (GEO) database respectively. Immune and metabolism related genes were collected from Immport and Kyoto encyclopedia of genes and genomes (KEGG) database respectively. Unsupervised consensus clustering was performed to identify the molecular subtypes. Cibersort was applied to evaluate the immune cells infiltration status. Differential expression analysis and Gene set enrichment analysis (GSEA) were performed to characterize the molecular pattern of different subtypes. Multivariate Cox regression analysis was used for prognosis prediction model construction and receiver operating characteristic (ROC) curve was used for performance evaluation. The hub genes in the model were verified in single-cell sequencing dataset and clinical specimens. *In vitro* experiments were performed to validate the findings in our research.

**Results:** Three subtypes were identified with prognostic implications. C1 subgroup was in an immunosuppressive state with activation of mitochondrial cytochrome P450 metabolism, C2 had poor immune cells infiltration and was characterized by tRNA anabolism, and the C3 subgroup was in an inflammatory state with activation of aromatic amino acid synthesis. The area under the ROC curve of the constructed model was 0.8, which showed better performance than clinical features. IMPDH1 was found to be significantly upregulated in tumor tissue and it was demonstrated that IMPDH1 could be a novel therapeutic target *in vitro*.

**Discussion:** In summary, our findings suggested novel molecular subtypes of cervical cancer with distinct immunometabolic profiles and uncovered a novel therapeutic target.

## Introduction

Cervical cancer (CC), a malignancy associated with high-risk human papillomavirus (HPV), remains the second leading cause of death in women aged 20–39 despite widespread early screening and prophylactic vaccination. It was demonstrated that a total of 4,152 women died of CC in 2019 (Siegel et al., 2022). It has been observed that there are significant individual differences in the prognosis of cancer patients despite controlling for factors such as age and clinical stage, which is likely due to tumor heterogeneity (Punt et al., 2017). For example, polymorphisms of TLRs that play a key role in innate immunity were found to be correlated with susceptibility to cervical cancer (Pandey et al., 2011). However, the tumor heterogeneity has not been fully clarified and the prognostic effect of the International Federation of Gynecology and Obstetrics (FIGO) staging system does not meet the clinical needs (Kupets and Covens, 2001). Therefore, it is necessary to explore the molecular heterogeneity and establish a more complete prognostic evaluation system, which may help improve the precision treatment for cervical cancer.

Cancer cells tend to be in the spotlight of many studies on cancer biology. However, it has been confirmed that the tumor microenvironment (TME) is a non-negligible factor in tumorigenesis, in which cancer cells may interact with extracellular matrix (ECM) and stromal cells. The TME is composed of a variety of cells, including fibroblasts, endothelial cells, mesenchymal cells and immune cells (Hanahan and Coussens, 2012). During interactions with tumor cells, the phenotype of both stromal cells and immune cells can be shaped to support tumor cell growth (Coussens et al., 2013). In the context of choric inflammation caused by cancer cells, myeloid cell precursors may be induced to proliferate and differentiate into the myeloid derived suppressor cells (MDSCs) with the binding of soluble tumor necrosis factor to the corresponding receptor (Sobo-Vujanovic et al., 2016). Once differentiated, MDSCs may be home to the TME and the subsequent vicious cycle of chronic inflammation, immunosuppression, tumor growth and differentiation cannot be stopped, which may result in poor prognosis (Ugel et al., 2015). Tumor associated macrophages (TAMs) were abundant in solid tumors and their appearance promoted tumor cell invasion and metastasis (Qian et al., 2009; Qian et al., 2011). It was demonstrated that the differentiation states of macrophages can be influenced by cancer cells (Mantovani and Sica, 2010). For example, Yang Cheng et al. found that PKN2 derived from colon cancer cells can inhibit M2 phenotype polarization, which may help promote anti-tumor immune response and improve prognosis (Cheng et al., 2018). CD8^+^ T cells play an important role in tumor suppression but they can be exhausted during the progression of cancer. Yongshuai Jiang et al. found that PRMT5 derived from cancer cells could suppress the function of tumor infiltrating T cells and promote the development of cervical cancer (Jiang et al., 2021). In addition, dendritic cells (DCs) expressing PD-1 were found to be correlated with advanced stages, elevated preoperative squamous cell carcinoma antigen levels and lymph-vascular space invasion, which suggests its role in immune surveillance dysfunction in cervical cancer (Wang et al., 2022). Overall, tumor-infiltrating immune cells were one of the important factors for the survival and prognosis of cancer patients, but the heterogeneity of TME in cervical cancer has not been fully elucidated and deserves further exploration.

It is well known that metabolic reprogramming is another characteristic of malignancies and plays an important role in tumor progression. Otto Warburg found that tumor cells generate energy through glycolysis under aerobic conditions and produce a lot of lactic acid, which shapes a hypoxic and acidic tumor microenvironment (Koppenol et al., 2011). Cytotoxic T cells are the most important cells in the anti-tumor immune response and their function was restricted by the glucose metabolism of tumor cells, resulting in tumor progression (Chang et al., 2015). It was shown that tumor glycolysis impacted T cell infiltration in the TME and impaired the efficacy of adoptive T cell therapy (Cascone et al., 2018). Aerobic glycolysis in tumor cells has been shown to promote the infiltration of MDSCs, thereby suppressing anti-tumor immune responses in triple-negative breast cancer (Li et al., 2018). On the other hand, immunomodulatory cells like M2 macrophages and regulatory T cells may impact the function of T cells by depleting the arginine in the TME (Speiser et al., 2016). Glutamine metabolism in tumor cells was enhanced in the TME where aerobic glycolysis produced large amounts of lactate, which caused glutamine deprivation to infiltrated immune cells and affected their proliferation (Carr et al., 2010). Tryptophan metabolism was found to play a role in immunosuppression state in various tumors (Platten et al., 2019). IDO1, one of tryptophan metabolizing enzymes, was demonstrated to correlate with low tumor infiltration of T cells in colorectal cancer (Brandacher et al., 2006), ovarian cancer (Inaba et al., 2009), and endometrial cancer (Ino et al., 2008). High levels of IDO and TDO have also been shown to contribute to impaired anti-tumor immune responses (Munn et al., 2005). Luc Pilotte et al. found that activation of tryptophan metabolism-related enzyme TDO2 in tumor cells can significantly inhibit the activation of T cells (Pilotte et al., 2012). Mutations of the isocitrate dehydrogenase genes IDH1 and IDH2 were found in the lower-grade glioma (LGG) and the mutated forms could convert α-ketoglutarate (α-KG) to the oncometabolite R-2-hydroxyglutarate (2HG) (Ichimura, 2012). Kohanbash G et al. found that IDH-MUT glioma reduced the production of T cells attracting chemokines and the accumulation of T cells was suppressed (Kohanbash et al., 2017). Therefore, tumor metabolic status can have an impact on infiltrating immune cells and may be a target for improving the efficacy of tumor immunotherapy.

In order to explore the heterogeneity of the TME in cervical cancer, an immune-metabolism related gene set was constructed and was applied to the identification of subgroups with prognostic significance in cervical cancer from The Cancer Genome Atlas Project (TCGA). Immune cell infiltration and metabolism status were evaluated respectively and the interaction was explored. Besides, we constructed a prognostic model in cervical cancer and validated the expression of key genes on a single-cell dataset from Gene Expression Omnibus (GEO). Our findings suggested the existence of immuno-metabolic subgroups of cervical cancer and uncovered novel therapeutic targets for cervical cancer.

## Methods and materials

### Human cervical cancer cohorts

The gene expression data of cervical cancer and the corresponding clinical information in the Cancer Genome Atlas (TCGA) were downloaded from the UCSC data portal (https://xenabrowser. net), which consists of 306 cervical tumor samples and 3 normal samples. We used the corresponding annotation file from the same database to convert the transcriptome raw count value in TCGA cohort to transcripts per kilobase million (TPM) values.

### Construction of the immunometabolism gene set and identification of the subtypes

851 immune related genes were downloaded from the Immport database (https://www.immport.org/home) and 1,401 metabolism related genes were downloaded from the Kyoto Encyclopedia of Genes and Genomes (KEGG) database (https://www.genome.jp/kegg). We applied the univariate Cox proportional hazards model to evaluate the association of these genes with overall survival. We included the genes with HR < 0.8 or HR > 1.2 and *p* values < 0.05 in the subsequent sample clustering. ConsensusClusterPlus, an R package designed for unsupervised consensus clustering, was used to identify the subclusters of the TCGA-CESC cohort.

### Evaluation of differences in gene expression and metabolism pathways among the subtypes

R (4.2.1) was used to identify the differentially expressed genes (DEGs) among the cervical cancer subtypes with the EdgeR package. Genes with an absolute value greater than 2 and an FDR value less than 0.05 were considered as DEGs. To clarify the pathway status among subtypes, Gene set variation analysis (GSVA) was performed with GSVA package to calculate the enrichment score of different pathways in every single CESC sample, including “hallmark gene set,” “KEGG gene set,” “GO biological processes,” “GO cellular components” and “GO molecular functions” downloaded from the Molecular Signatures Database (MsigDB, https://www.gsea-msigdb.org/gsea/msigdb).

### Evaluation of the immuno-metabolic microenvironment

Cibersort, a deconvolution algorithm for dissecting the cell component in bulk sequence data, was used to characterize the abundance of various immune cells infiltrated and the corresponding cell states (Newman et al., 2015). As for the evaluation of difference in metabolic status, 85 metabolic pathways and the corresponding gene sets were acquired from the KEGG database and the single sample gene set enrichment analysis (ssGSEA) algorithm was used to calculate the enrichment scores. Subsequently, Limma package was used to perform the differential analysis of the metabolic pathways.

### Generation of the prognostic gene signature

The entire cohort was randomly divided into a training dataset (70%) and a validation dataset (30%). In the training cohort, the Lasso-Cox regression analysis was used to select the genes with prognostic value, Then, a multivariate Cox hazard ratio model was constructed with 10 genes selected. The risk score was calculated based on the expression data in the validation cohort and the corresponding coefficients in the model. According to the coefficients in the model, the formula for calculating the risk score is: risk score = FLT3LG*(−0.147) + IMPDH1*(-0.011) + OPRD1*0.836 + MOCS1*0.163 + IL1B*0.331 + GALNT10*0.489 + TNFRSF11B*(−0.048) + LDHC*(−1.075) + ISG20*(−0.444) + TRAV12_3*(−0.134). Finally, We used the survivalROC package to evaluate the prognostic value. With the median value of risk score, we divided the entire cohort into high and low risk groups, for which survival analysis was performed.

### Gene expression analysis at single cell resolution

To elucidate the cell specificity of gene expression and validate the difference between tumor and normal samples. GSE168652, a single cell sequencing data of cervical cancer derived from Hua’s research (Li et al., 2021) was downloaded from Gene Expression Omnibus (GEO) datasets. Seurat (version 4.1.1) was used to perform quality control, data filtration, data scale, dimension reduction, clustering and cell type annotation, in which the criteria were set the same as the original research. For pseudo-time trajectory analysis, Monocle (version 2.20.0) was used to analyze the cell state transition of cancer cells and visualize the gene expression patterns along the trajectory.

### Immunohistochemistry

Human cervical cancer tissue sections were retrospectively obtained from surgical resections that were fixed in buffered formalin, embedded in paraffin, and stored at the Zhujiang Hospital, Southern Medical University, Guangzhou, China. The corresponding clinical data were obtained from medical records and identified. The ethics committee of Zhujiang Hospital approved the use of the clinical specimens. For IHC staining, antigen retrieval was performed by heat treatment in a microwave oven for 21 min in Tris-ethylene diamine tetraacetic acid (EDTA) buffer solution (0.05 mol/L Tris, 0.001 mol/L EDTA; pH 8.5). Endogenous peroxidase activity was inactivated using 0.3% H_2_O_2_ for 10 min followed by washing with PBS (Gibco, C14190500BT). After blocking by 5% BSA for 20 min, the slides were incubated overnight at 4°C with the following primary antibodies used (proteintech, 22092-1-AP-50UL). After washing with PBS, the sections were incubated with HRP conjugated goat anti-rabbit IgG secondary antibodies (Cell Signalling, 7074) for 50 min. Finally, immunoreactivity was detected using 3,3-diaminobenzidine (Servicebio, G1211), followed by re-staining with hematoxylin. Images were obtained by using 3D HISTECH (Pannoramic MIDI II).

### Cell culture

Human cervical cancer cell lines HeLa, SiHa, Caski and c33a were purchased from the Procell Life Science&Technology Co.,Ltd. Cells were cultured in Dulbecco’s modified Eagle’s medium (DMEM) with supplement of 10% fetal bovine serum (FBS) (Gibco, 10099-141C) and 100 units/mL penicillin and streptomycin (Sigma, St. Louis, MO, United States) at 37°C with a humidified atmosphere of 5% CO_2_ maintenance.

### Western blotting

Cells were homogenized in RIPA lysis buffer (sc-24948; Santa Cruz Biotechnology, Inc.), and protein contents were measured using Bicin-choninic Acid (BCA) protein assay kit (CWBIO, CW0014S). Incubate cell protein with SDS-PAGE loading buffer (CWBIO, CW0052S) at 100°C for 10 min to denature the protein. After electrophoresis with SDS-PAGE, the separated proteins from the gel were transferred onto polyvinylidene fluoride (PVDF) membrane and then were subjected to western blotting with specific primary antibodies followed by detection with horseradish peroxidase-conjugated secondary antibody (solarbio, SE134-1mL, SE131-1 mL) and enhanced chemiluminescence (Merck millipore, WBKLS0100). The antibodies used in this study include the following: anti-IMPDH1 antibody (proteintech, 22092-1-AP-50UL, 1:2000); GAPDH (MC4) Mouse Monoclonal Antibody (Beijing Ray Antibody Biotech, RM 2002, 1:50000). ImageJ software was used to quantify the protein bands. Target protein expression was normalized to GAPDH to correct for loading.

### Quantitative real-time PCR

Total RNA from cells was isolated by TRIzol extraction according to the manufacturer’s instructions (Thermo Fisher, 15596026), and cDNA was synthesized with a reverse-transcription kit (Vazyme, R323-01). The quantitative real-time PCR (qRT-PCR) experiment was conducted using SYBR Green Real-Time PCR Master Mix Kit (Vazyme, Q711-02) with the Light Cycler LC480 (Roche). Primer pairs for quantitative real-time PCR were synthesized from Tsingke Biotechnology Co., Ltd. IMPDH1(F: 5′-CAG​CAG​GTG​TGA​CGT​TGA​AAG-3′, R: 5′-AGC​TCA​TCG​CAA​TCA​TTG​ACG-3′); ACTB(F: 5′-AGA​GCT​ACG​AGC​TGC​CTG​AC-3′, R: 5′-AGC​ACT​GTG​TTG​GCG​TAC​AG-3′) Values were calculated by the change in threshold method (ΔΔCT).

### CCK8

Cells were seeded at 5,000 cells per well in 96-well plates according to the manufacturer’s guidelines. Cells were allowed to adhere overnight, and treated with different interventions (*n* = 3 wells/group) for the indicated time. A total of 10 μL Cell Counting Kit-8 (CCK8) reagents (APExBIO, K1018-5) were added and incubated for 1 h. Then, the absorbance was read at 450 nm. Statistical analysis (mean ± SD) with triplicates is shown.

### Flow cytometry

After induction of apoptosis, cells from each treatment condition were washed once in PBS. The apoptotic rate was evaluated according to the protocols provided by the Annexin V-FITC Apoptosis Detection Kit (Beyotime, China). Generally, 1 × 10^5 cells were diluted within buffer, and stained with FITC-conjugated Annexin V and PI according to the manufacturer’s instructions. The cell mixture was cultured at room temperature for 20 min and then analyzed by the CytoFLEX instrument (Beckman).

### Transfection

For knockdown assays, short interfering RNAs (siRNAs) targeting IMPDH1 were synthesized by RiboBio Co., Ltd. (Guangdong, China). The sequence are listed: genOFFTM st-h-IMPDH1_001: 5′-GGT​GAT​GAC​GCC​AAG​GAT​T-3′; genOFFTM st-h-IMPDH1_002: 5′-GCA​CCG​ACC​TGA​AGA​AGA​A-3′; genOFFTM st-h-IMPDH1_003: 5′-GTA​CAA​GGT​GGC​TGA​GTA​T-3′; All cells were transfected using Lipofectamine 3000 Reagent (Invitrogen, Carlsbad, CA, United States) according to the manufacturer’s instructions.

### Statistical analysis

Cox regression model was used to evaluate the hazard ratio and prognostic significance of genes in the OS. KM and Cox regression analysis were applied to calculate the significance of difference in OS, PFS and DSS. Log-rank test was used to evaluate the statistical difference of the KM curves. For evaluation of the predictive power of immuno-metabolism risk score to OS, the time-dependent area under the receiver operating characteristic curve (AUC) and C-index (also termed concordance index) were calculated. Higher value of these two indicators represented better accuracy. In terms of correlation analysis, the Spearman method was used to calculate the correlation coefficient and the *p*-value. Kruskal–Wallis test was used when the statistical difference of distribution in three or more groups was examined and Wilcoxon test was used when comparisons contain only two groups. If not specified, *p* values were two-sided and *p* < 0.05 was defined as statistically significant.

## Results

### Identification of the immune-metabolism subtypes of cervical cancer

**FIGURE 1:**
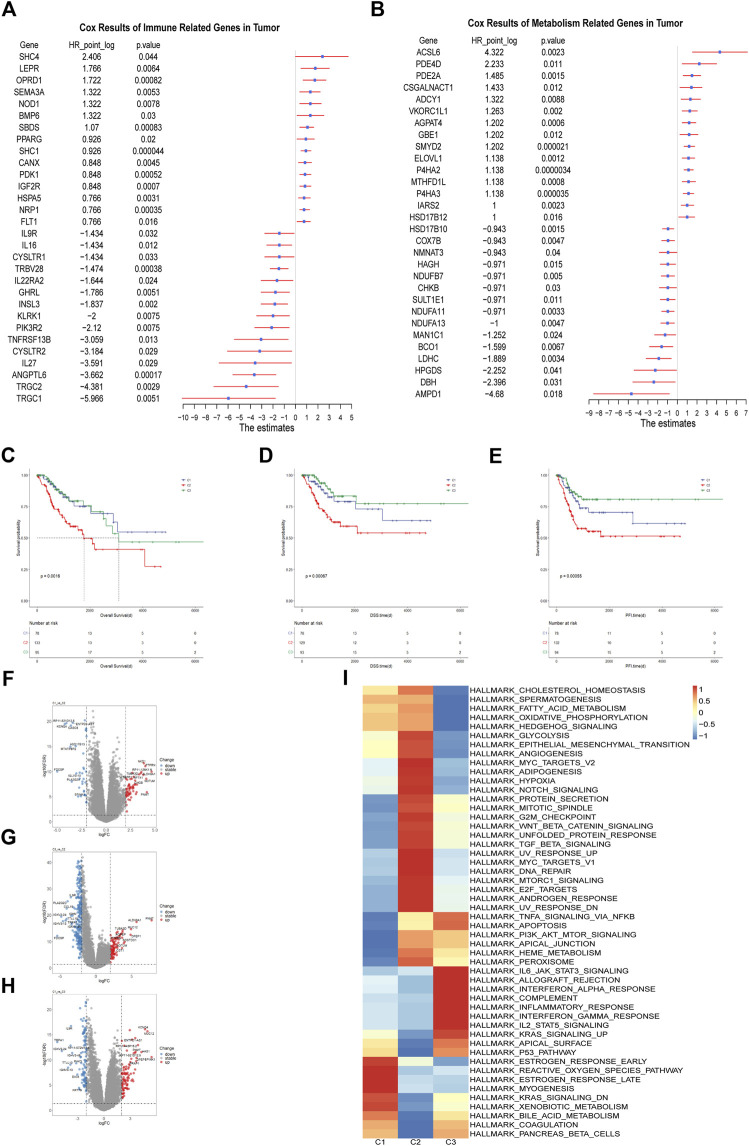
Identification of the Immune-metabolism Subtypes of Cervical Cancer **(A)** Hazard ratio of top 30 immune related genes that meet the requirements of HR < 0.8 or HR > 1.2 and *p* < 0.05 associated with overall survival. **(B)** Hazard ratio of top 30 metabolism related genes that meet the requirements of HR < 0.8 or HR > 1.2 and *p* < 0.05 associated with overall survival. **(C–E)** Kaplan-Meier curves of overall survival, progression free interval and disease specific survival among the subtypes in TCGA cohort. **(F–H)** The volcano plots of differentially expressed genes among the three subtypes in TCGA cohort. **(I)** Heatmap of differentially enriched hallmark pathways from GSEA database among three subtypes in TCGA cohort.

The immune related genes and the metabolism related genes were downloaded from the Immport database and KEGG database respectively. We constructed an immune-metabolism gene set and determined the candidate genes with prognostic values using Univariate Cox proportional hazards regression model analysis. As a result, the top three immune related genes with unfavorable prognosis were SHC4(HR = 5.3, *p* = 0.044, 95%CI, 1–26), LEPR (HR = 3.4, *p* = 0.0064, 95%CI, 1.4–8.1) and OPRD1(HR = 3.3, *p* = 0.00082, 95%CI, 1.6–6.6) while TRGC1(HR = 0.016, *p* = 0.0051, 95%CI, 0.00092–0.29), TRGC2(HR = 0.048, *p* = 0.0029, 95%CI, 0.0065–0.35) and ANGPTL6(HR = 0.079, *p* = 0.00017, 95%CI, 0.021–0.3) were the top three significant protective factors (Figure 1A). It was reported that SHC4 was involved in the progression of hepatocellular cancer (Urabe et al., 2020) and prostate cancer (Zhang et al., 2022). LEPR was found to be overexpressed in epithelial ovarian cancer indicating poor progression-free survival (Uddin et al., 2009) and its somatic mutation was found to increase the susceptibility to hepatocarcinogenesis (Ikeda et al., 2014).

In terms of metabolism, we identified ACSL6 (HR = 20, *p* = 0.0023, 95%CI, 2.9–140), PDE4D (HR = 4.7, *p* = 0.011, 95%CI, 1.4–16) and PDE2A (HR = 2.8, *p* = 0.0015, 95%CI, 1.5–5.2) as the top three risk factors. AMPD1 (HR = 0.039, *p* = 0.018, 95%CI, 0.0027–0.57), DBH (HR = 0.19, *p* = 0.031, 95%CI, 0.043–0.86) and HPGDS (HR = 0.21, *p* = 0.041, 95%CI, 0.047–0.94) were identified as the top three protective factors (Figure 1B). PDE4D was demonstrated to be a tumor-promoting factor in prostate cancer (Rahrmann et al., 2009) and BRAF-mutated melanoma (Delyon et al., 2017). Inhibition of PDE4D helped to overcome tamoxifen resistance in ER-positive breast cancer (Mishra et al., 2018).

Those genes with HR > 1.2 or HR < 0.8 and *p* < 0.05 in the Cox regression model were selected for the subsequent clustering analysis, which contained 154 immune-related genes and 195 metabolism related genes. We applied the unsupervised cluster analysis for the dataset and it was indicated that the entire cohort could be divided into three subgroups (Supplementary Figures 1A–1F). Survival analysis showed that overall survival (OS), progression free survival (PFS) and disease specific survival (DSS) differed significantly among these three subgroups. The C2 subgroup showed worst prognosis (Figures 1C–E). There were distinct gene expression patterns among the three subpopulations so we analyzed the differentially expressed genes (DEGs) among them (Figures 1F–H). Finally, we identified 97 DEGs between C1 and C2, 197 DEGs between C1 and C3 and 337 DEGs between C2 and C3. To explore the difference in signal pathway status, we performed GSVA analysis in the three cervical cancer subgroups. It was indicated that C2 was characterized by some cancer-related pathways such as angiogenesis, hypoxia, and epithelial-mesenchymal transition while C3 was shown to have inflammatory signature, with inflammatory response, interferon gamma response and reactive oxygen species pathway significantly upregulated. As for C1 subgroup, it was characterized by estrogen response, KRAS signaling and xenobiotic metabolism activation (Figure 1I). It is well known that the rapid progression of malignancy may create a hypoxic microenvironment and hypoxia can stimulate the expression of some angiogenesis related factors, which may exacerbate tumor immunosuppression and adversely affect patient outcomes (Rahma and Hodi, 2019). Taken together, We identified three subgroups of cervical cancer with unique molecular features and prognostic significance.

### Characterization of the immune microenvironment among different subpopulations

**FIGURE 2:**
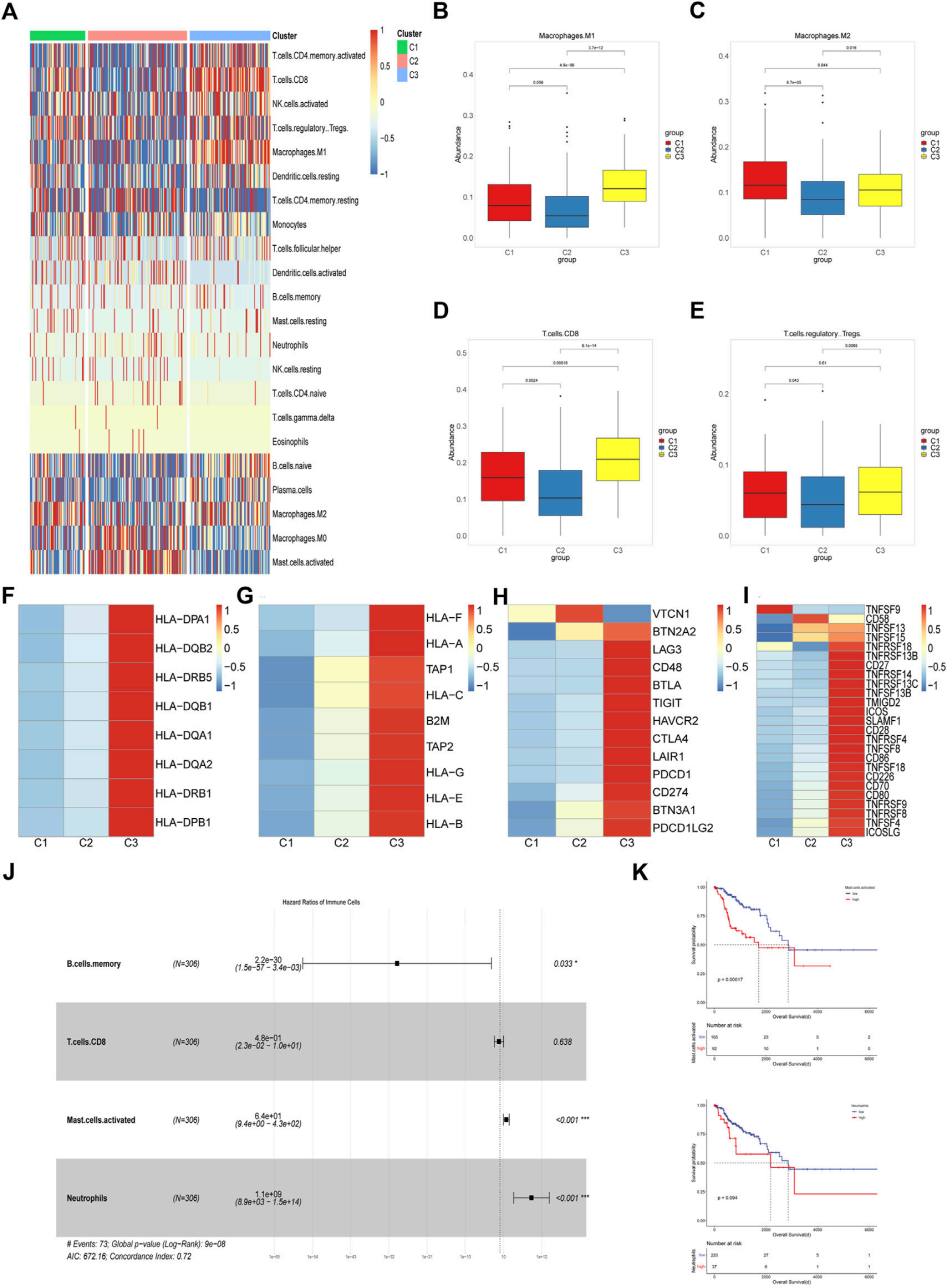
Characterization of the Immune Microenvironment Among Different Subpopulations **(A)** Heatmap of the abundance of 22 immune cells among three subgroups in TCGA cohort. **(B–E)** Boxplots of abundance of M1 macrophages, M2 macrophages, CD8^+^ T cells and Tregs among three different subtypes in TCGA cohort. The differences were compared using the Kruskal–Wallis test. **(F,G)** Heatmap of differential expression of MHC molecules among three subtypes in TCGA cohort. **(H,I)** Difference of co-stimulatory and co-inhibitory molecules expression level in three subtypes. **(J)** Multivariate cox regression model constructed based on the immune cells with prognostic value in univariate cox analysis. **(K)** Kaplan-Meier curves of overall survival between cohorts in TCGA with high and low abundance of activated mast cells and neutrophils infiltrated.

Emerging evidence showed that solid tumor harbor rather complex components, which included immune cells, fibroblasts, endothelial cells and mesenchymal cells and tumor cells. Tumor microenvironment plays an important role in cancer development, immune escape and metastasis (Quail and Joyce, 2013). Different immune microenvironment components were closely related to the responsiveness of chemotherapy, immunotherapy and patient prognosis (Liu et al., 2022). With Cibersort, we evaluated the abundance of 22 immune cells infiltrated in the three subgroups. It was shown that most immune cells infiltrated poorly in the C2 subgroup while abundantly in the C3 subgroup, moderately in the C1 subgroup, which indicated that C3 subgroup had better anti-tumor immune response than others (Figure 2A).Pandey et al. (2009) found that TLRs, the pathogen recognition receptors mainly expressed on immune cells, were correlated with susceptibility to cervical cancer. Therefore we analyzed the expression of TLR2, TLR3 and TLR4 among three subtypes and found that C3 had the highest level, which may account for better innate immunity and better prognosis in the C3 subgroup (Supplementary Figures 2A–2C). Anti-tumor immune response includes multiple steps and various kinds of immune cells were involved in it such as dendritic cells, B cells, macrophages, nature killer cells, and T cells (Motz and Coukos, 2013). Macrophages can be influenced by tumor cells to differentiate into M1 or M2 subtypes, wherein M1 is a pro-inflammatory and anti-tumor subtype while M2 is an anti-inflammatory and tumor-promoting subtype. We compared the abundance of macrophages among the three subgroups and found that C2 subpopulation had the lowest abundance of both subtypes of macrophages while C1 and C3 were dominated by M2 and M1 macrophages respectively (Figures 2B, C). CD8^+^ T cells are an important part of anti-tumor immunity but are prone to depletion phenotype transformation in the tumor microenvironment, which is one of the reasons for the low response rate of tumor immunotherapy. We found that CD8^+^ T cell abundance was highest in the C3 subpopulation, lowest in the C2 subpopulation, and intermediate in the C1 subpopulation, which may indicate that C2 has the worst anti-tumor immune status (Figure 2D). As for regulatory T cells, both C1 and C3 were significantly more abundant than C2 while there was no significant difference between C1 and C3, suggesting that C1 has immunosuppressive characteristics (Figure 2E). Antigen presentation is an important step in immune response, which is dependent on the expression of major histocompatibility complex (MHC). It was reported that cancer cells can evade attack by immune cells through downregulating the expression of MHC molecules (Marincola et al., 2000; van der Burg et al., 2016). So we evaluated the expression of several MHC I/II molecules and found that C1 and C2 subpopulations showed lower expression level than C3 subpopulation (Figures 2F, G). Activation and expansion of T cells require the co-stimulatory molecules and we found that C1 and C2 had significantly lower levels of co-stimulatory molecules compared to C3 (Figure 2H). In addition, high levels of co-inhibitory molecules were detected in the C3 subpopulation, which may indicate that C3 subpopulation benefits from immune checkpoint blockade (ICB) therapy (Figure 2I). It is well known that cytotoxic T cells depend on interferon gamma (IFN-γ) and granzyme B to attack cancer cells (Jenkins and Griffiths, 2010). In our study significantly lower expression level of these two genes and lower enrichment score of IFN-γ signaling were found in C1 and C2, suggesting impaired T cells function in these two subpopulations (Supplementary Figures 2D–2E). Finally, we assessed the prognostic significance of various immune cells in the cohort. Multivariate cox regression analysis showed that the abundance of memory B cells was an independent protective factor and that activated mast cells and neutrophils were independent risk factors (Figure 2J). Survival analysis showed that the population with higher mast cell abundance had a significantly worse prognosis than the population with lower mast cell abundance while neutrophil abundance did not significantly distinguish the cohort for survival differences (Figure 2K).

Overall, we identified three subgroups of the cervical cancer cohort based on the immune-metabolism gene set and analyzed the immune infiltration status of the three subgroups. We found that C3 had the highest abundance of immune cell infiltration and was characterized by inflammation, C2 had the least abundance of immune cells and had the worst prognosis, and C1 had some immune cell infiltration but mainly had an immune-exhausted phenotype.

### Metabolic characteristics of cervical cancer subgroups

It was known that malignancies undergo metabolic reprogramming in order to adapt to the needs of rapid proliferation (Martínez-Reyes and Chandel, 2021), resulting in abnormal accumulation of metabolites in the solid tumor microenvironment, thereby affecting various cellular components in the tumor microenvironment (Li et al., 2019).

**FIGURE 3:**
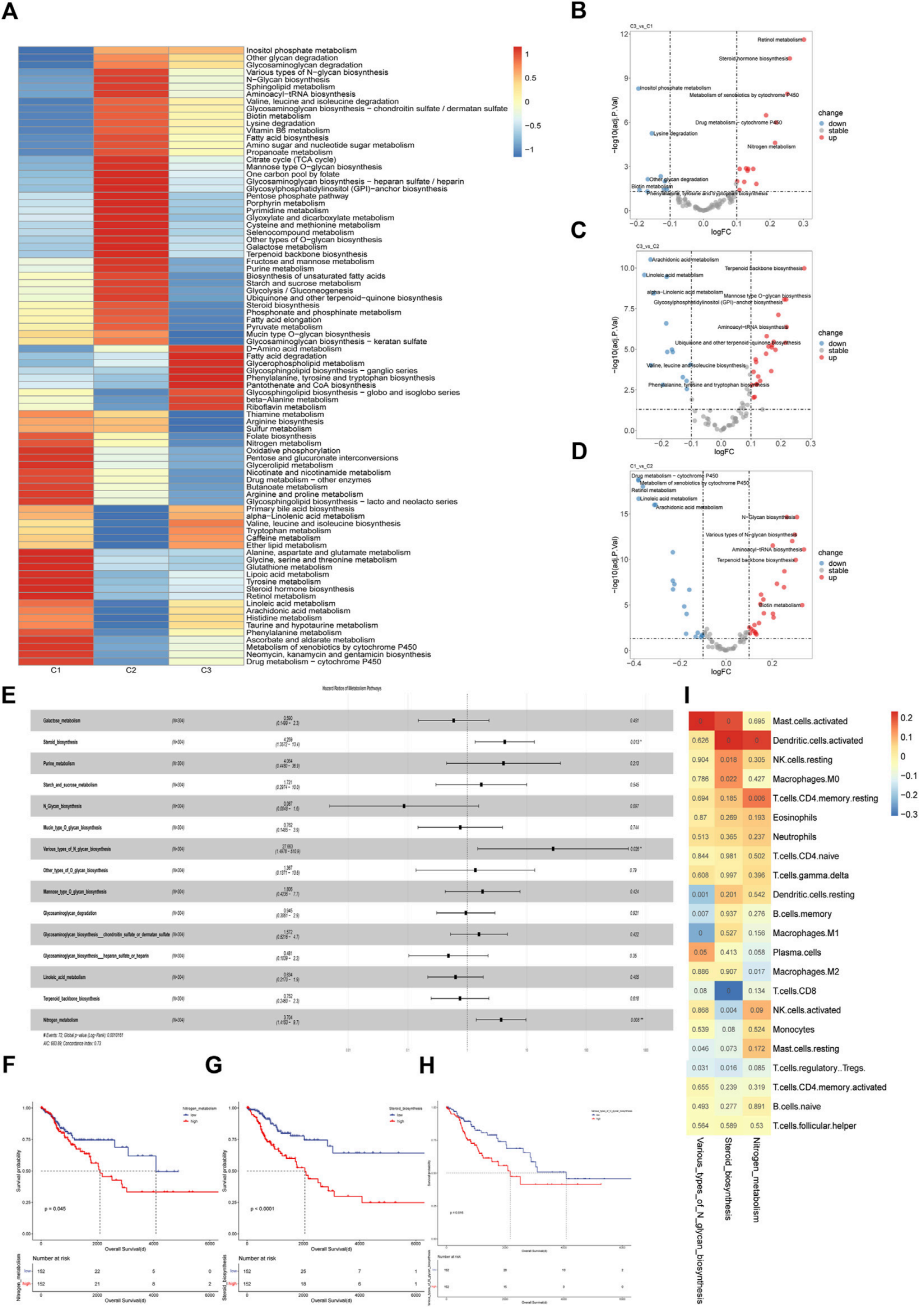
Metabolic characteristics of cervical cancer subgroups **(A)** Heatmap of differentially enriched metabolic pathways in three subtypes in TCGA cohort. The enrichment analysis was performed with GSVA algorithm. **(B–D)** Volcano plot of differentially enriched metabolic pathways among three subtypes in TCGA cohort. The differential analysis was performed based on the GSVA analysis. **(E)** Multivariate cox regression model constructed based on the metabolic pathways with prognostic value in univariate cox analysis. **(F–H)** Kaplan-Meier curves of overall survival between cohorts in TCGA with high and low enrichment score of nitrogen metabolism, steroid biosynthesis and various types of N glycan biosynthesis. The enrichment analysis was performed with GSVA algorithm. **(I)** Correlation matrix of the specific metabolic pathways and the 22 immune cells infiltrated in the whole cohort. Correlation coefficients are represented in the form of heatmap using colored scale ranging from blue (minimum correlation) to red (maximum correlation) and the *p*-value were presented.

Using the GSVA algorithm, we performed an enrichment analysis of 85 metabolic pathways in the KEGG database for the cervical cancer cohort. The enrichment score heatmap showed a distinct pattern in metabolic status among the three subgroups (Figure 3A). Next, we performed differential analysis of metabolic pathways among the three subgroups and the results showed that a total of 107 differential metabolic pathways were identified. After ranking by logFC value, the top 5 pathways were included in subsequent analysis, and only those upregulated in comparison with other subgroups were considered significantly enriched pathways. It was illustrated that the drug metabolism-cytochrome P450 pathway, metabolism of xenobiotics by cytochrome P450 pathway and retinol metabolism pathway were significantly upregulated in the C1 subpopulation (Figure 3B). The metabolic pathways that were significantly upregulated in C2 were aminoacyl-tRNA biosynthesis pathway and terpenoid backbone biosynthesis pathway (Figure 3C). As for C3, phenylalanine, tyrosine and tryptophan biosynthesis were most significantly upregulated (Figure 3D). To explore the prognostic significance of multiple metabolic pathways in the whole cohort, we performed a univariate cox analysis for each metabolic pathway, and only those metabolic pathways with statistical significance were included in the subsequent multivariate cox regression analysis. The results showed that steroid biosynthesis, various type of N-glycan biosynthesis and nitrogen pathway were independent risk factors in multivariate cox regression analysis (Figure 3E). Based on the median of these three metabolic pathways enrichment scores, we divided the cohort into groups with high and low levels of the corresponding pathways, and then performed survival analysis. We found that higher activity of the three metabolic pathways was associated with worse prognosis (Figures 3F–H). Next, we performed a correlation analysis between immune cell abundance and the enrichment scores of metabolic pathways with independent prognostic value (Figure 3I). The data showed that both nitrogen metabolism (*r* = 0.22, *p* < 0.01) and steroid biosynthesis (*r* = 0.23, *p* < 0.01) pathway were significantly positively correlated with activated dendritic cell. M1 macrophages were significantly negatively correlated with the various type of N-glycan biosynthesis pathway (*r* = −0.24, *p* < 0.01) while M2 macrophages were significantly negatively correlated with the nitrogen metabolism pathway (*r* = −0.14, *p* = 0.017). The abundance of CD8^+^ T cells (*r* = −0.31, *p* < 0.01) and activated NK cells (*r* = −0.16, *p* = 0.004) showed significantly negative correlation with steroid biosynthesis pathway. Regulatory T cells (Tregs) are significantly negatively correlated with the various type of N-glycan biosynthesis (*r* = −0.12, *p* = 0.031) and steroid biosynthesis pathway (*r* = 0.14, *p* = 0.016). According to the published research, it was demonstrated that metabolic reprogramming in tumor cells could impair the anti-tumor immunity (Hung et al., 2021; Kao et al., 2022). Therefore, it was indicated that correlation and interaction existed between tumor cells and infiltrated immune cells in cervical cancer. The phenotype and function of immune cells may be affected by tumor metabolic reprogramming, which may promote immune escape in CC.

### Construction of an immuno-metabolic prognostic model for cervical cancer

**FIGURE 4:**
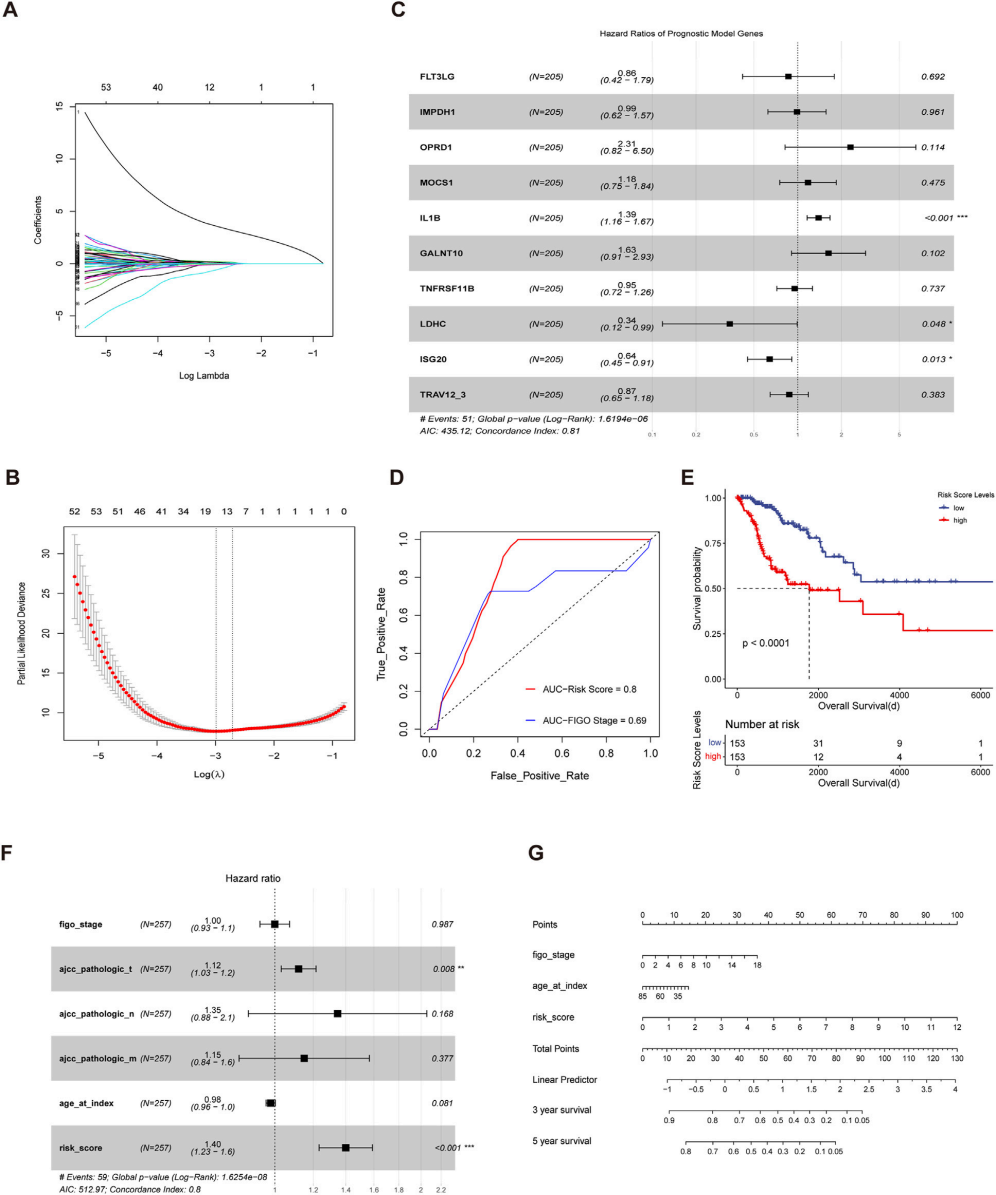
Construction of an immuno-metabolic prognostic model for cervical cancer **(A,B)** Fit and cvfit plots of LASSO screen. **(C)** Forest plot of Multivariate cox regression model constructed based on the genes screened out from LASSO in training cohort. **(D)** ROC curves measuring the predictive value of risk score and clinical stage. The area under the ROC curve was 0.82 and 0.69 for the risk score and clinical stage, respectively. **(E)** Kaplan-Meier curves of overall survival between the cohorts of TCGA with low and high risk score. **(F)** Forest plot of the multivariate cox regression model constructed with clinical stage, age and risk score. **(G)** Nomogram for predicting probability of survival at 3 and 5 years in cervical cancer.

Based on the immune-metabolism gene set, we identified patient subgroups with significantly different prognosis. Therefore, the immunometabolism gene set was applied to construct a cervical cancer prognosis prediction model. Firstly, we divided 70% of the cervical cancer cohort into the training cohort and the remaining 30% into the validation cohort. Then, with the lasso-cox regression method, we screened the 10 genes with the prognostic value from the immune metabolism gene set (Figures 4A, B), which included FLT3LG, IMPDH1, OPRD1, MCOS1, IL1B, GALNT10, TNFRSF11B, LDHC, ISG20 and TRAV12-3. Next, a multivariate cox regression model based on the 10 prognostic-related genes was constructed in the training cohort and the risk scores were calculated based on the corresponding gene coefficients in the model (Figure 4C). With the risk scores, we performed a prognostic prediction in the validation cohort and the area under the receiver operating curve of the prediction model was 0.8, which was higher than that of the clinical stage prediction model of 0.69 (Figure 4D). Based on the median risk score, the whole cohort was divided into a high-risk group and a low-risk group, and there was a significant difference in survival between the two groups (*p* < 0.0001) (Figure 4E), indicating that immuno-metabolic factors were important for the prognosis of cervical cancer patients. Additionally, a multivariate cox regression analysis incorporating immuno-metabolic risk model scores with FIGO stage, TMN stage, and age was performed, and risk score was found to be an independent adverse prognostic factor (Figure 4F). Finally, a nomogram for prognosis prediction was constructed for cervical cancer (Figure 4G).

### IMPDH1 was a significant prognostic risk gene in cervical cancer

**FIGURE 5:**
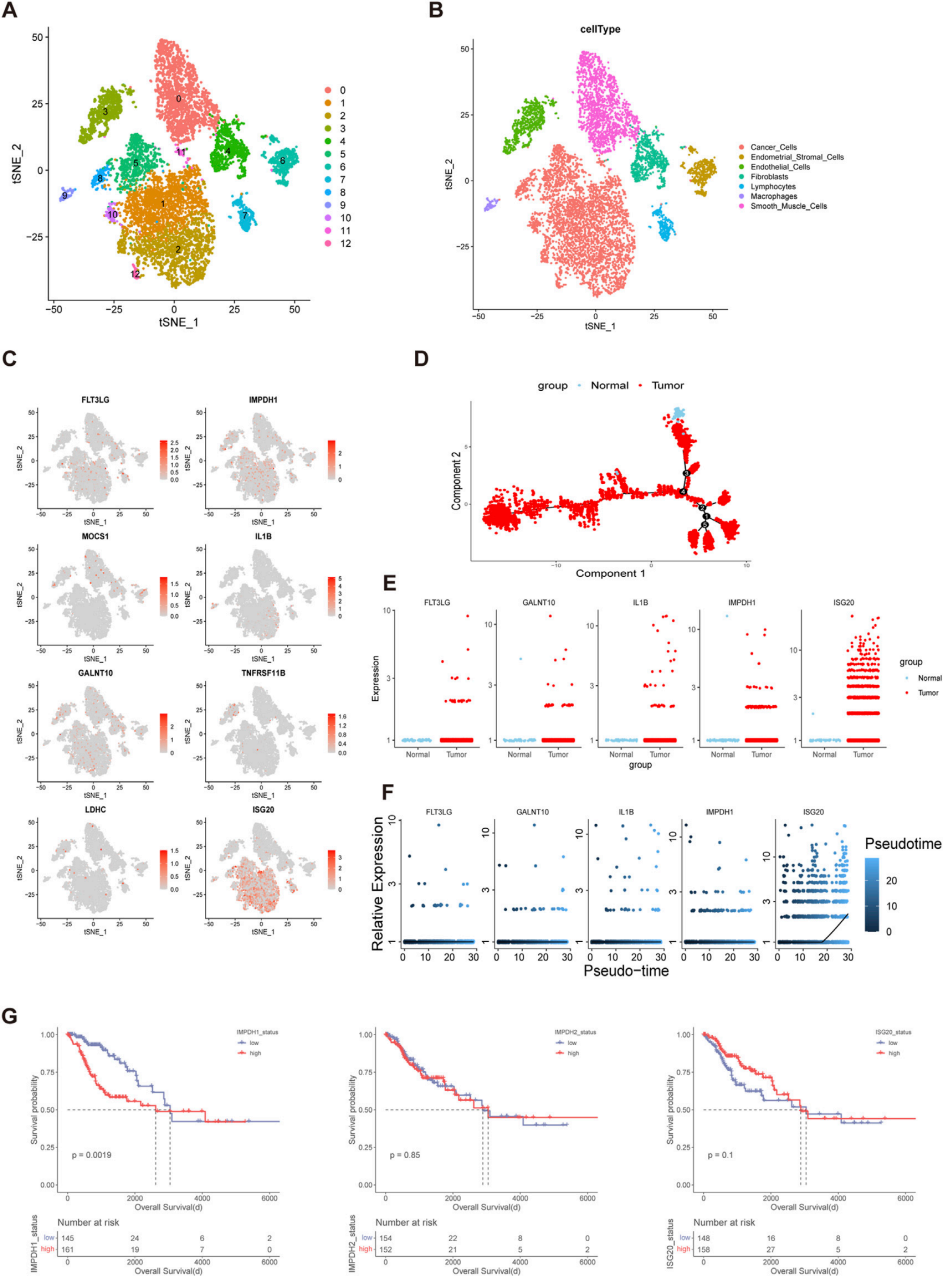
IMPDH1 was A Significant Prognostic Risk Gene in Cervical Cancer **(A,B)** The t-distributed stochastic neighbor embedding (t-SNE) plot demonstrating the main cell clusters in cervical cancer and identification of the main cell types. **(C)** Heatmap shows the expression of genes in the risk models. **(D)** Development trajectory plot of cervical cancer cells in pseudo-time analysis. **(E)** Differential expression of the genes involved in the risk model between the normal cervix cells and cervical cancer cells. **(F)** Pseudo-time analysis showing the expression patterns of the genes in the risk model along the tumor progression. **(G)** Kaplan-Meier curves of overall survival of the cohorts with low and high expression level of IMPDH1, IMPDH2 and ISG20 in TCGA.

Bulk sequencing failed to distinguish the effects of cellular components in the TME on tumorigenesis while single-cell sequencing can make up for this limitation. To verify the cellular origin of genes in prognostic models, a single-cell transcriptome dataset of cervical cancer was downloaded from Gene Expression Omnibus (GEO) database and used for analysis. Data filtration, integration, and dimensionality reduction clustering were performed according to the parameter in original research. A total of 13 cell clusters were identified and cancer cells, endometrial stromal cells, endothelial cells, fibroblasts, lymphocytes, macrophages, and smooth muscle cells were annotated respectively according to corresponding cell surface markers (Figures 5A, B). IMPDH1 and ISG20 were mainly expressed in tumor cells, while the remaining genes were less specific to tumor cell origin (Figure 5C). Then we performed a pseudo-time analysis of tumor cells and compared the expression differences of these genes between normal cells and tumor cells (Figure 5D). The results showed that the expression levels of FLT3LG, GALNT10, IL1B, IMPDH1 and ISG20 were higher in tumor cells than in normal cells, while the remaining genes could not be identified because of the low expression levels in tumor cells (Figure 5E). In addition, we found that with the evolution of tumor cell status, the expression levels of FLT3LG, GALNT10, IL1B, and IMPDH1 remained stable while ISG20 gradually increased (Figure 5F). It was shown that IMPDH1 was one of the isoforms of inosine-5′-monophosphate dehydrogenase (IMPDH) which contributed to the formation of cytoophidia and tumor progression (Ruan et al., 2020a). For further analysis of IMPDH1, IMPDH2 and ISG20, we divided the TCGA cohort into high and low level groups based on the mean expression levels of corresponding molecules. The results showed that there were significant prognostic differences between groups with different expression levels of IMPDH1 but not IMPDH2 or ISG20 (Figure 5G).

### *In vitro* validation

**FIGURE 6:**
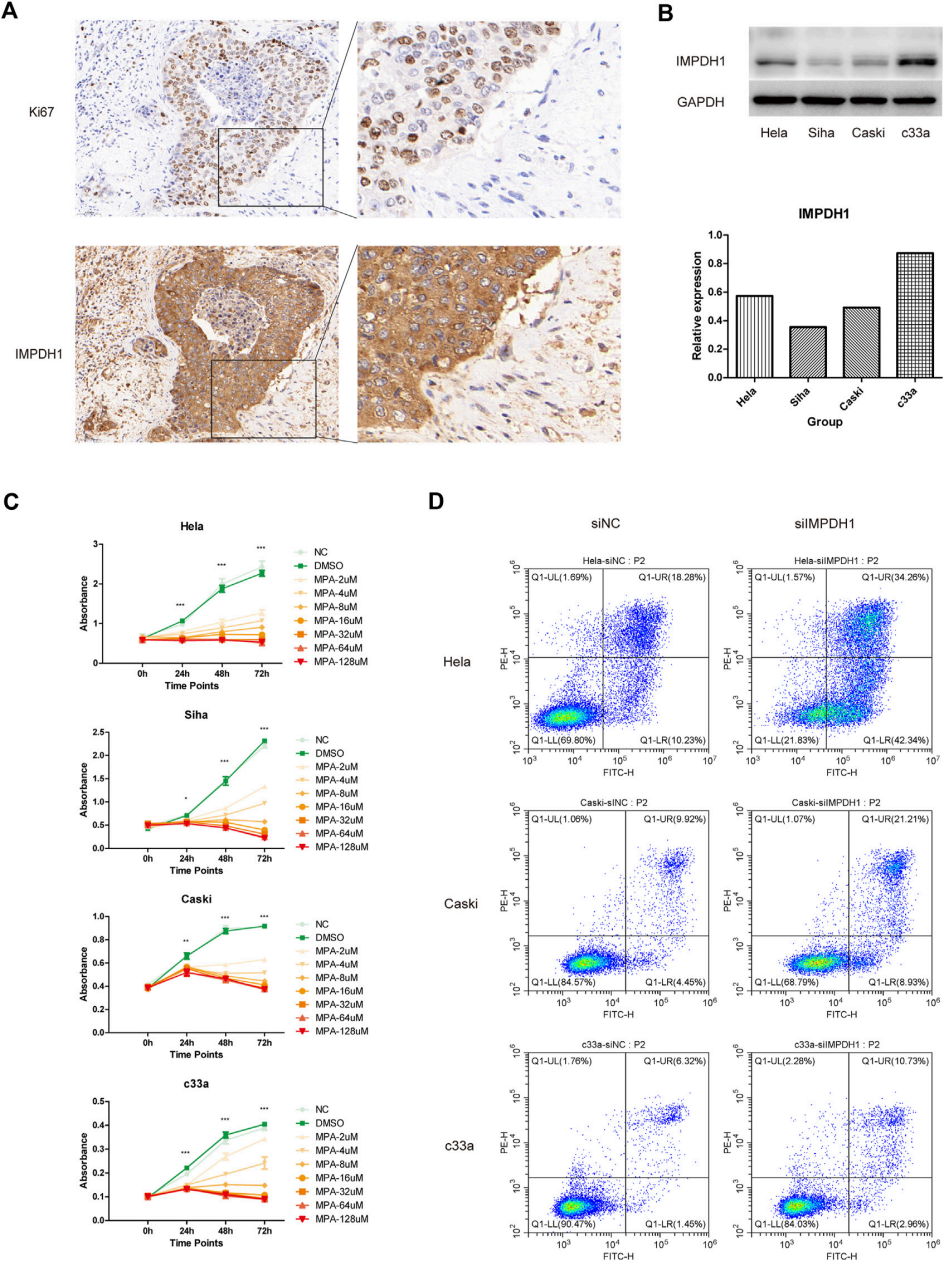
*In vitro* validation **(A)** IMPDH1 protein expression in normal cervix tissue and cervical cancer determined using immunohistochemistry. **(B)** Different expression level of IMPDH1 in Hela, Siha, Caski and c33a cell lines. **(C)** CCK8 assay of cell viability under intervention at different concentrations of MPA. **(D)** Flow cytometry analysis of apoptosis rate of cells transfected with siRNA. *p < 0.05, **p < 0.01, ***p < 0.001.

It was found at single cell resolution that IMPDH1 was mainly expressed in cervical cancer cells. Therefore we validated our finding on clinical specimens with immunohistochemistry. Compared with normal cervix tissue, the expression of IMPDH1 in cervical cancer was significantly increased, suggesting IMPDH1 may contribute to the progression of cervical cancer (Figure 6A). Then we examined the expression level of IMPDH1 in four cervical cancer cell lines Hela, Caski, c33a and Siha with western blotting. It was shown that 4 cell lines have different expression level (Figure 6B). Mycophenolic acid (MPA) was a pan-inhibitor of IMPDH and it was demonstrated that targeting IMPDH with MPA can significantly inhibit growth of the ASCL1^low^ small cell lung cancer cell (Huang et al., 2018). In our research, we found that the cell viability of cervical cancer cell lines could also be significantly inhibited with MPA, which indicated IMPDH could be targeted in cervical cancer (Figure 6C). In addition, siRNA was used to knock down the expression of IMPDH1 in Hela, Caski and c33a cell lines. Western blotting and qRT-PCR confirmed the knockdown efficiency (Supplementary Figures 3A–3C). With flow cytometry, we found that the apoptosis rate was higher in cells transfected with siRNA (Figure 6D). Taken together, IMPDH1 may contribute to the growth of cervical cancer and it can be a novel therapeutic target in cervical cancer.

## Discussion

In the present study, we identified three molecular subgroups with distinct prognosis in cervical cancer from immuno-metabolic perspective. The differences in tumor infiltrating lymphocytes and metabolic characteristics among the three subgroups were evaluated with Cibersort algorithm and GSVA algorithm respectively. In addition, a risk model based on immune and metabolism related genes was constructed for prognosis prediction in cervical cancer, which showed higher accuracy than current FIGO stage in the validation cohort. With the single cell sequencing data from Hua’s research (Li et al., 2021), we explored the hub genes’ cellular localization and their expression patterns during cancer progression. Finally, we found that IMPDH1 may be a key gene in tumorigenesis, whose expression may help shape the TME and promote tumor progression.

Tumor heterogeneity is an important factor affecting the survival and prognosis of patients with cancer. Molecular stratification has been applied to multiple malignancies in order to help inform appropriate clinical decisions, including prostate cancer (Tang et al., 2022), breast cancer (Wolf et al., 2022), hepatocellular carcinoma (Molina et al., 2022), intrahepatic cholangiocarcinoma (Martin-Serrano et al., 2022) and small cell lung cancer (Rudin et al., 2019). It was demonstrated that multiple molecular subtypes of cervical cancer may be uncovered from different aspects (Meijer and Steenbergen, 2017). Integration analysis from The Cancer Genome Atlas Research Network revealed different molecular features of cervical cancer, which may help personalize clinical management (Cancer Genome Atlas Research Network et al., 2017). Maud Kamal et al. discovered the different integration signatures of HPV genome in cervical cancer that may imply prognostic significance (Kamal et al., 2021). With the 50 genes having the largest expression variation, Xiaojun Zhu et al. demonstrated two molecular subgroups in cervical cancer and explored the heterogeneity, which provided novel targets for diagnosis and treatment (Zhu et al., 2022). Here we provided a novel classification approach to dissect the heterogeneity of cervical cancer from the immunological and metabolic perspectives. Three immuno-metabolic subtypes were identified in cervical cancer with significantly different prognosis. The immune infiltration status was poorer in C2 subgroup than the other two subgroups and C2 had the worst prognosis. The expression level of immune checkpoint molecules was higher in the C3 subgroup, which suggested better response to immune checkpoint blockade therapy. Due to the limited number of samples, there was no significant prognostic difference between C1 and C3 subgroups but they showed distinct metabolism status, indicating novel therapeutic targets for tumor metabolism. Therefore, this stratification strategy may contribute to individualized treatment for cervical cancer.

Immune microenvironment was one of the most important facets of tumorigenesis (Schreiber et al., 2011). It was demonstrated that the abundance of tumor infiltrated lymphocytes (TILs) was associated with favorable patient prognosis (Jérôme et al., 2006). Consistent with the existing evidence, C2 subgroup showed the least immune cells abundance and therefore the prognosis was worst among the three subgroups. The phenotype of TILs can be shaped towards immunosuppressed by tumor cells during cancer progression. For example, macrophages infiltrating the tumor can be induced to differentiate into the M2 phenotype that tends to suppress immune response (Luca and Pollard Jeffrey, 2018). In our study, macrophages in C1 subgroup were characterized with anti-inflammatory phenotype while macrophages in C3 subgroup were pro-inflammatory. In terms of PFI and DSS, C3 subgroup showed better prognosis than C1 subgroup though the difference was not statistically significant. Therefore, we speculated that the status of immune cells may determine the prognosis of cervical cancer. It was shown that the lactate derived from tumor cells can influence the phenotype of macrophages in lung cancer and melanoma (Colegio et al., 2014). Our research showed that the glycolysis level is rather higher in C1 subgroup, which may explain the difference of macrophage differentiation between C1 and C3 subgroups. Besides, activated mast cells were found abundant in C2 subgroup and they indicated poor prognosis in the whole cohort, which was consistent with the existing evidence (Huang et al., 2008). Different expression level of TLRs was detected among the three subgroups, which indicated that distinct status of innate immunity against HPV may exist in cervical cancer patients and correlate with prognosis. Due to the data limitation, we cannot distinguish the expression level or polymorphism of TLRs in cancer cells in TCGA cohort and therefore the molecular mechanism by which TLRs promote cervical cancer progression needs further experimental research.

Distinct metabolic patterns were uncovered among the three immuno-metabolic subgroups. Compared with the other two subgroups, C2, the subgroup with the worst prognosis, was characterized with terpenoid backbone biosynthesis and aminoacyl-tRNA biosynthesis. 3-Hydroxy-3-methylglutaryl-CoA synthase 1 (HMGCS1), a metabolic enzyme that participated in terpenoid backbone biosynthesis, was demonstrated to involve in the progression of cervical cancer (Zhang et al., 2020a). Besides, Li et al. (2022) showed that isoprenylcysteine carboxyl methyltransferase (ICMT) may mediate the malignant development of cervical carcinoma. Other genes in terpenoid backbone biosynthesis pathway have been demonstrated to play a role in tumorigenesis of breast cancer (Yu et al., 2021), prostate cancer (Seshacharyulu et al., 2019) and renal cell carcinoma (Huang et al., 2021). During protein synthesis, aminoacyl-tRNA biosynthesis was an Indispensable pathway catalyzed by 20 essential enzymes that ligate the amino acids to their corresponding tRNAs and there was evidence that the synthetases may cause diseases when they were mutated or expressed abnormally (Kwon et al., 2019). For example, the expression of glycyl-tRNA synthetase (GRS) was found to be an indicator of unfavorable outcomes in renal, urothelial, liver, breast and endometrial cancers (Thul and Lindskog, 2018). In our research, we found a correlation between the metabolism pathway mentioned above and the rarity of TILs. Therefore we speculated that metabolism related genes may impact the abundance and status of TILs in addition to their involvement in tumor metabolic reprogramming, which deserves further exploration in cervical cancer.

Ten genes were used for the construction of the prognostic model, which included FLT3LG, IMPDH1, OPRD1, MOCS1, IL1B, GALNT10, TNFRSF11B, LDHC, ISG20 and TRAV12-3. This model showed better predictive power than FIGO stage system, suggesting its potential for clinical application. FLT3LG, the formative cytokine for cDC1, was shown to be capable of controlling the levels of type I conventional dendritic cells in TME and increasing the responsiveness of patients to anti-PD-1 immunotherapy (Barry et al., 2018). In contrast, we validated the expression levels of FLT3LG at single cell resolution and found that it was upregulated mainly in cancer cells, which indicated its distinct function in cervical cancer. IMPDH1 is a rate-limiting enzyme of guanosine triphosphate (GTP) *de novo* synthesis and it can also form a filamentous structure called cytoophidia (Keppeke et al., 2020). According to Ruan et al. (2020b)’s research, cytoophidia formed by IMPDH1 may contribute to the metastasis of clear cell renal cell carcinoma. Consistently, IMPDH1, but not IMPDH2, was found to be an indicator of poor prognosis in our cohort, suggesting that cytoophidia may be a therapeutic target in cervical cancer. Dysregulated Inflammation cytokine can exacerbate tumor development. MOCS1 is a gene involved in the molybdenum cofactor biosynthesis pathway (Reiss and Hahnewald, 2011) but little was known about its function in malignancies. IL1B was one of the IL-1 family proteins and it was demonstrated to be a therapeutic target in cancer (de Mooij et al., 2017). Altered glycosylation was found to occur in malignancy (Oliveira-Ferrer et al., 2017) and GALNT10 is one of the glycosyltransferases whose expression was associated with poor prognosis in high grade ovarian serous cancer (Zhang et al., 2020b). TNFRSF11B, also termed osteoprotegerin, was demonstrated to involve in the progression of gastric cancer (Luan et al., 2020), melanomas (Oliver et al., 2013) and colon cancer (Zhang et al., 2021) but little was known about its function in cervical cancer. LDHC is one of the isozymes in lactate dehydrogenase family which catalyzes the interconversion of pyruvate and l-lactate (Markert et al., 1975). Remy Thomas et al. demonstrated that LDHC could be a targetable cancer antigen for cancer immunotherapy (Thomas et al., 2020). ISG20 is a 20 kDa protein that was capable of inhibiting multiple viruses and its expression was shown to contribute to poor survival in glioma (Gao et al., 2019). Surprisingly, some genes in the model were not detected at single cell resolution such as OPRD1, a gene encoding the delta-opioid receptor and TRAV12-3, a gene encoding the T cell receptor alpha variable, which may be due to the sample heterogeneity of single cell sequencing. Besides, TRAV12-3 served as a protective factor in the model and it suggested a role for robust immunity in preventing HPV-related tumors.

There were some limitations existing in our research. Firstly, the identification of tumor subtypes was based on the analysis of the public data without further exploration in experiment or clinical investigation. Second, with limited data resources, the construction and verification of our prognostic model were performed in different parts of one cohort, which needs further validation in external cohort. Finally, bioinformatics methods were used to evaluate the prognostic prediction power of 10 key genes in cervical cancer but their underlying molecular mechanisms in the tumorigenesis and progression deserve further research. Though the clinical specimens and experimental data showed that IMPDH1 can be a therapeutic target in cervical cancer, more detailed mechanism needs further research.

Taken together, our research provided a novel perspective for molecular stratification in cervical cancer. Distinct metabolic patterns were found in three subgroups and the correlation between TILs and metabolic pathways were explored. Then a risk model for prognostic prediction was constructed and it showed better performance than clinical stage. Immune-metabolism risk score exhibited unfavorable prognostic significance. Finally, we verified the genes in model at single cell resolution to figure out the cellular localization and found that five genes were significantly upregulated in cervical cancer cells. These findings will help adjust the management strategy for cervical cancer according to their heterogeneity and uncover novel targets for cancer immunotherapy.

## Data Availability

The datasets presented in this study can be found in online repositories. The names of the repository/repositories and accession number(s) can be found in the article/Supplementary Material.

## Supplementary material

SUPPLEMENTARY FIGURE S1 Results of unsupervised consensus clustering **(A)** Consensus matrix legend plot indicating the cluster probability ranging from 0 to 1 with different colors. **(B–D)** Consensus matrix heatmap of different k value (*k*=2, *k*=3, and *k*=4 respectively). **(E)** Cumulative distribution function (CDF) plot of consensus clustering. **(F)** Delta area plot of consensus clustering.

SUPPLEMENTARY FIGURE S2 Expression of TLRs and cytotoxicity related molecules and signaling. **(A-C)** Boxplot of the expression of TLR2, TLR3 and TLR4 among three subtypes. The differences were compared using the Kruskal-Wallis test. **(D–E)** Boxplot of the expression of grazyme B and IFN-γ among three subtypes. The differences were compared using the Kruskal-Wallis test. **(F)** Boxplot of the enrichment score of IFN-γ signaling among three subtypes. The differences were compared using the Kruskal-Wallis test.

SUPPLEMENTARY FIGURE S3 Validation of the knockdown efficiency of siRNA **(A-C)** Results of qRT-PCR and western blotting confirmed the knockdown efficiency of siRNA targeting IMPDH1.
