# Supplementary figures and images for: Characterization of the microenvironment in different immune-metabolism subtypes of cervical cancer with prognostic significance
Source: Front Genet. 2023 Feb 3;14:1067666. doi: 10.3389/fgene.2023.1067666 (PMC9935837; doi:10.3389/fgene.2023.1067666)

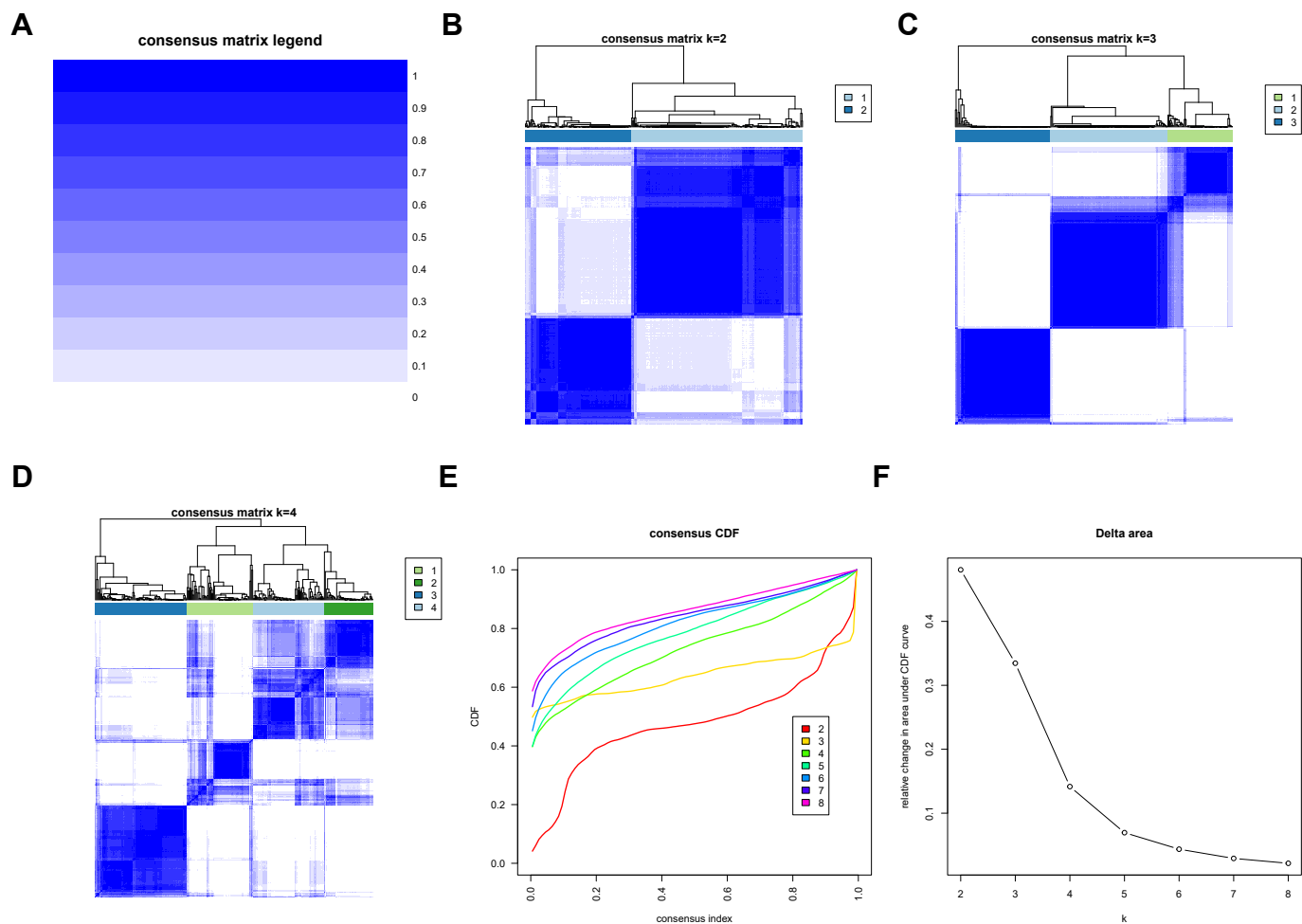

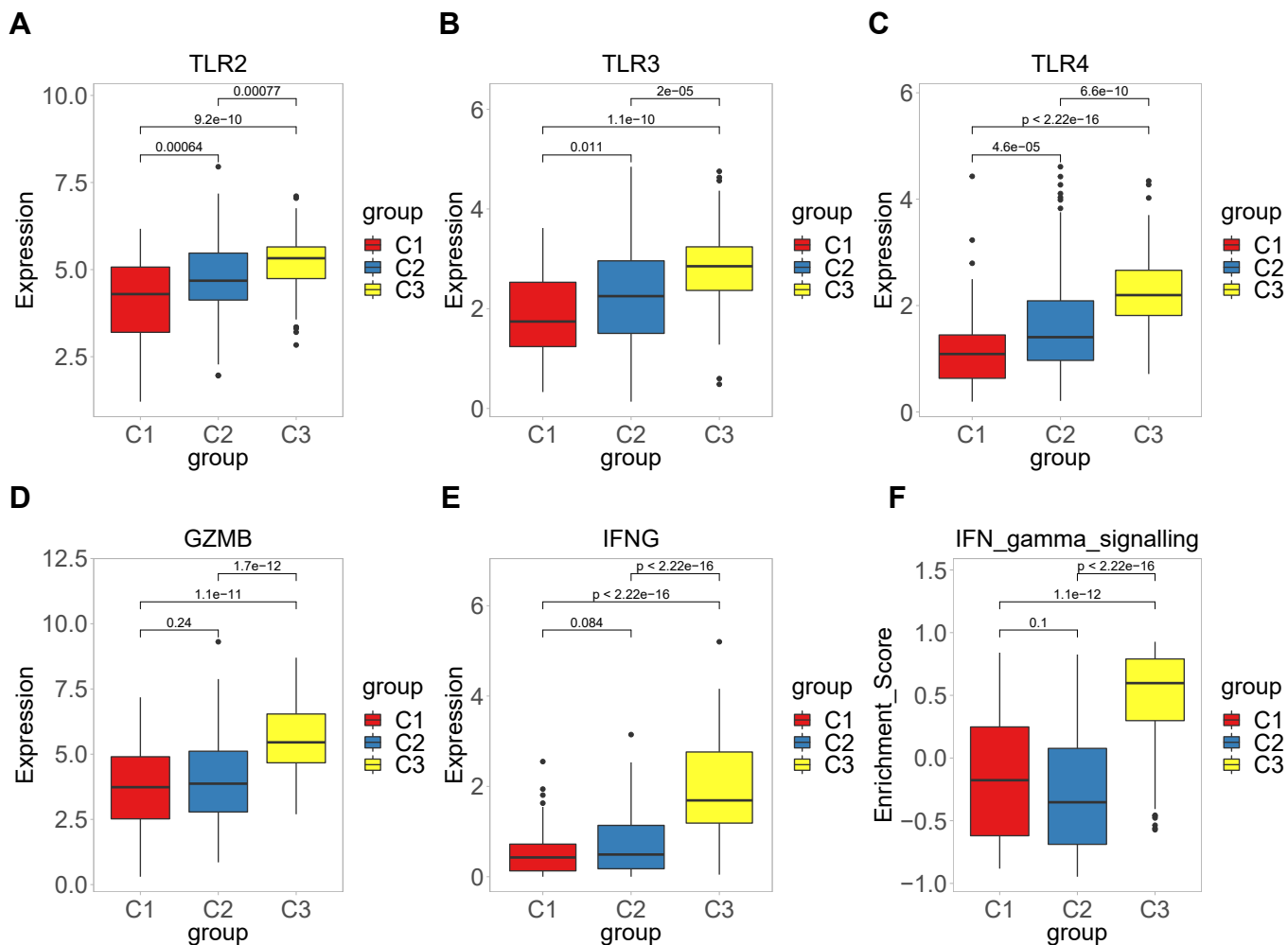

**A**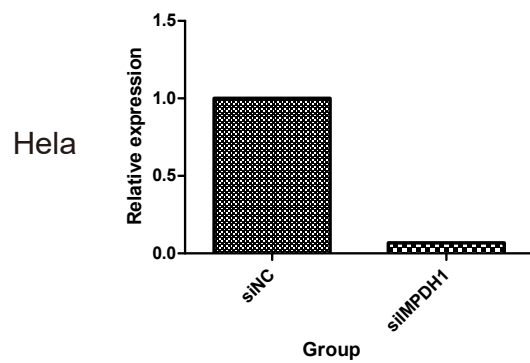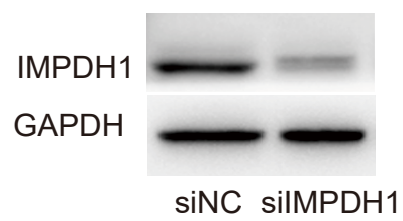**B**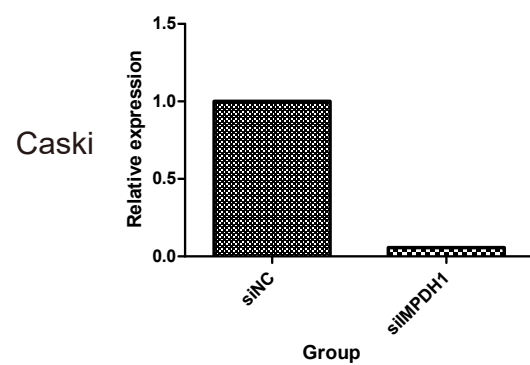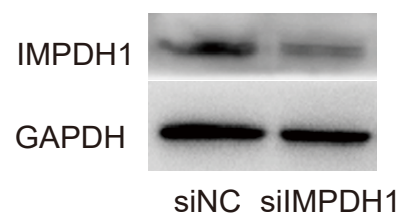**C**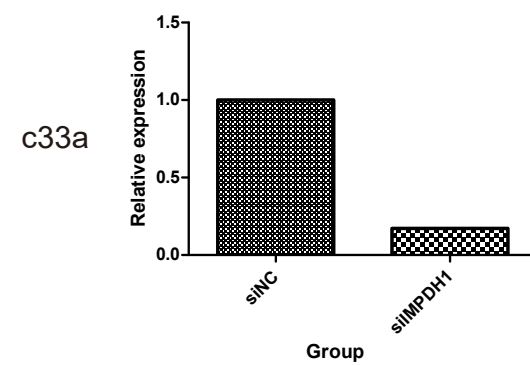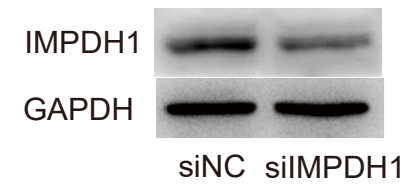

Supplement: Supplementary file 1 [file DataSheet2.PDF]

A

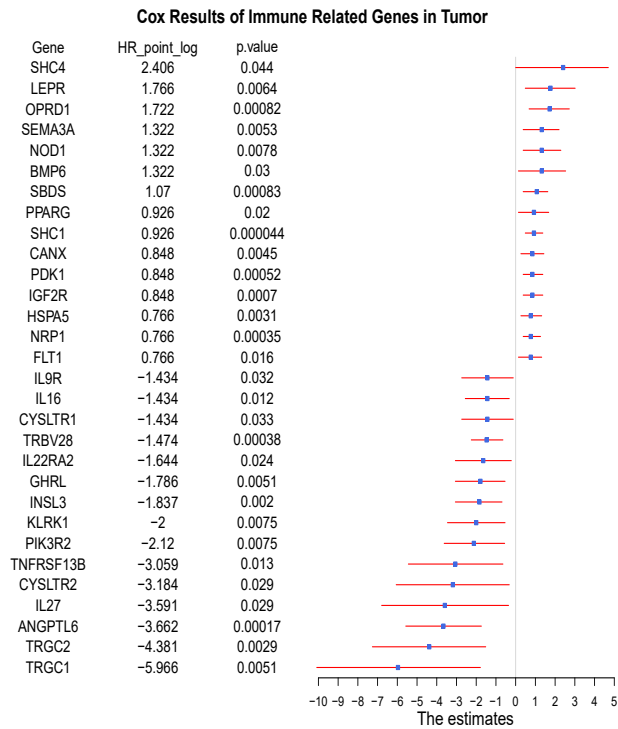

B

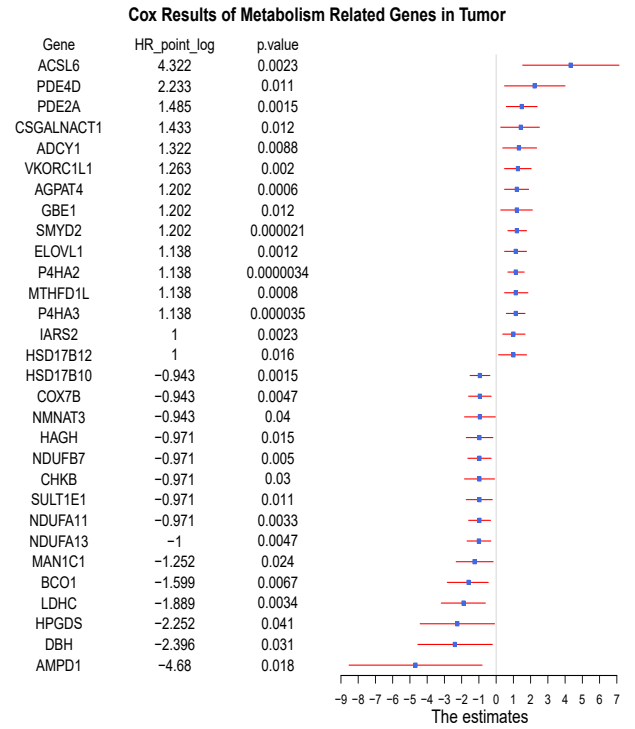

C

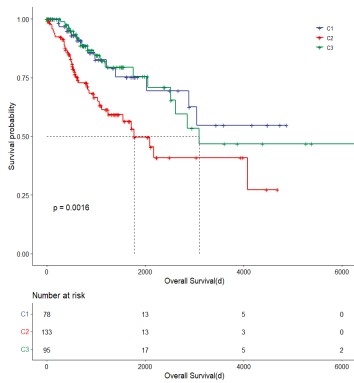

D

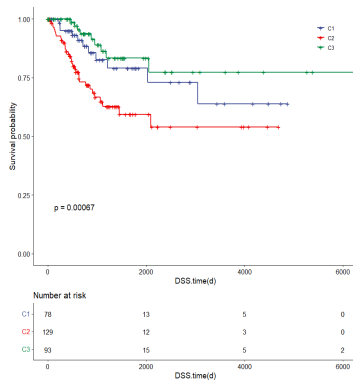

E

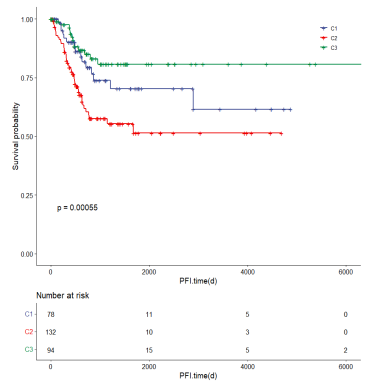

F

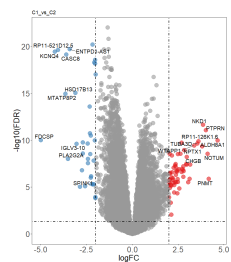

G

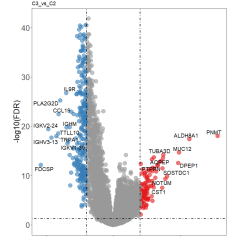

H

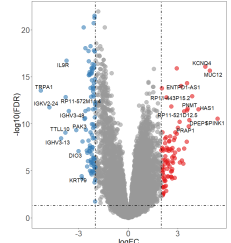

I

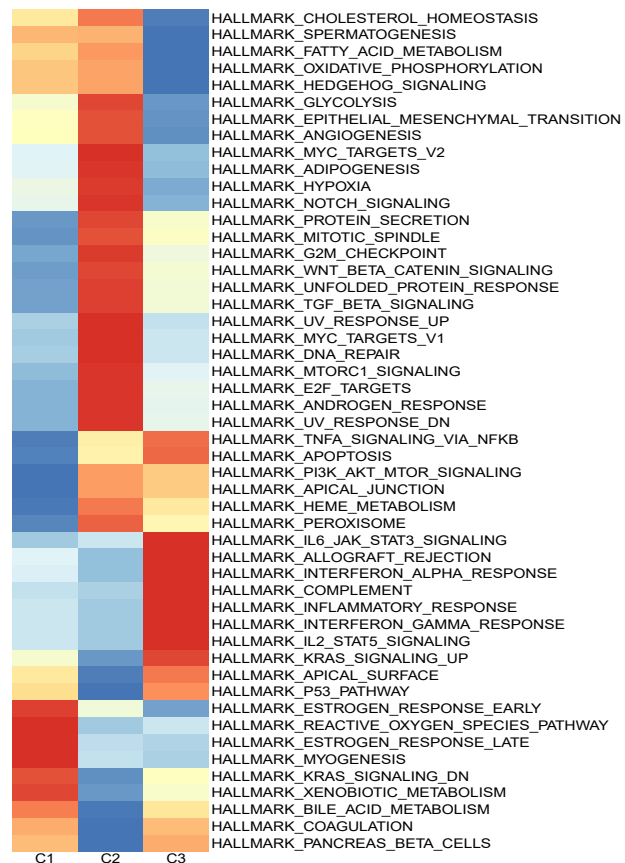

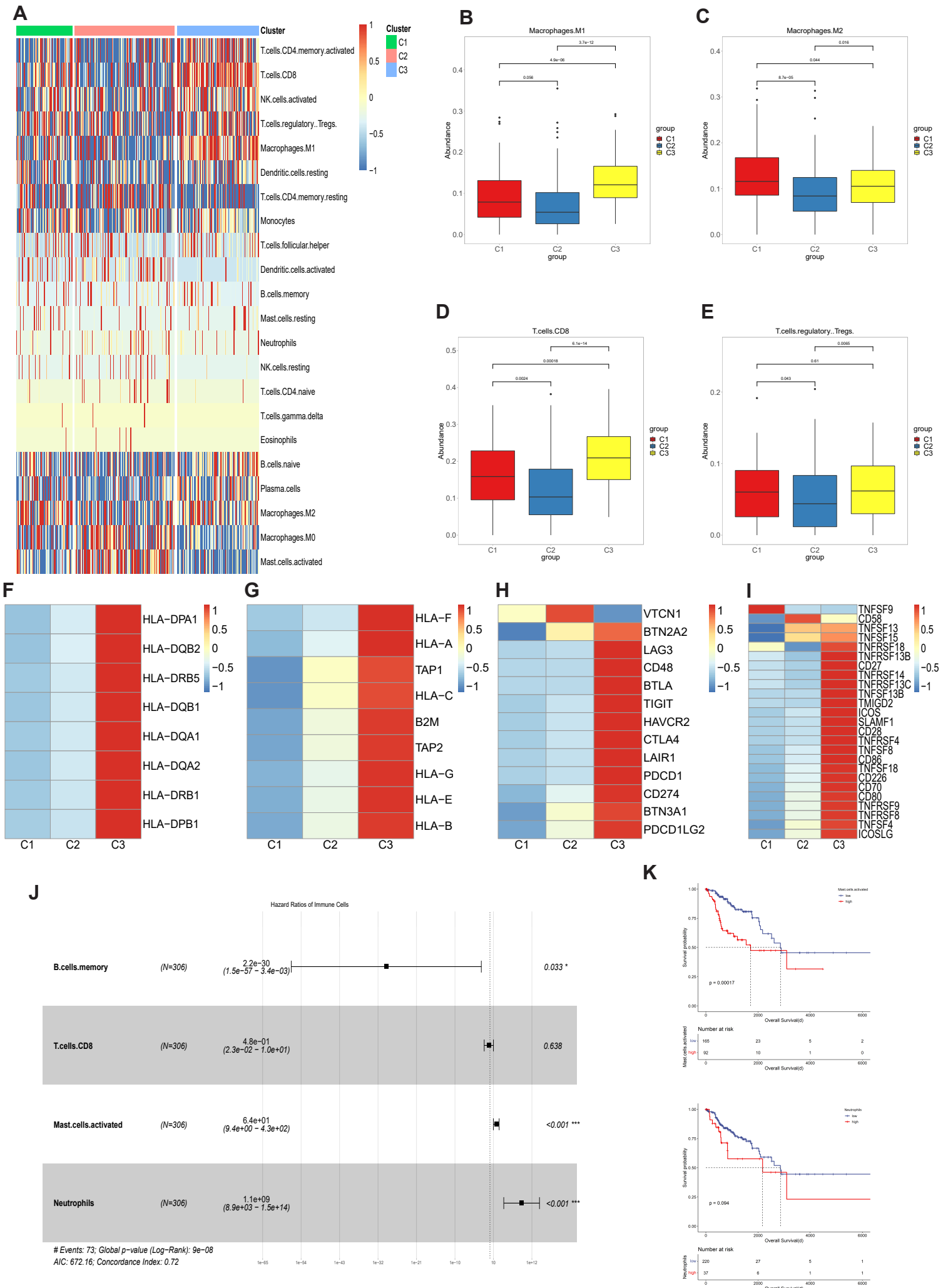

A

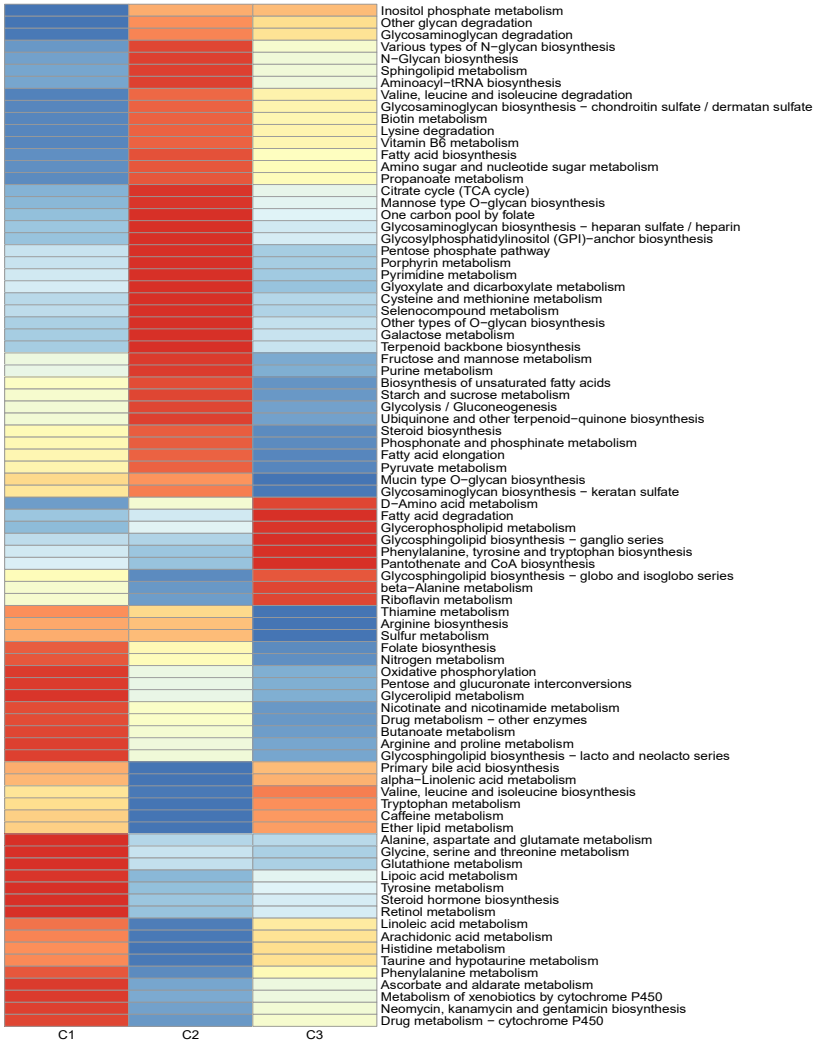

B

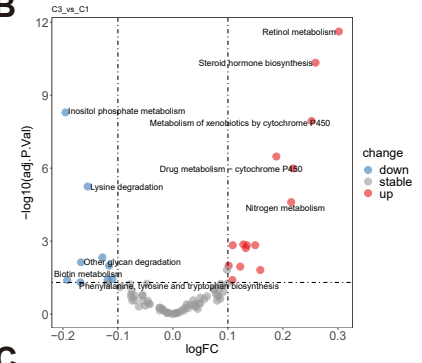

C

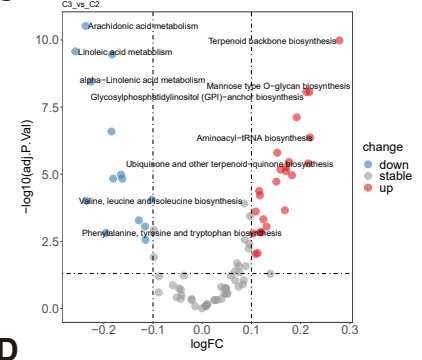

D

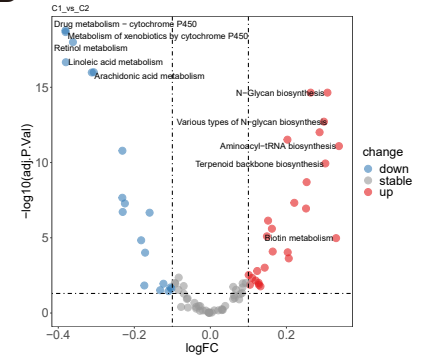

E

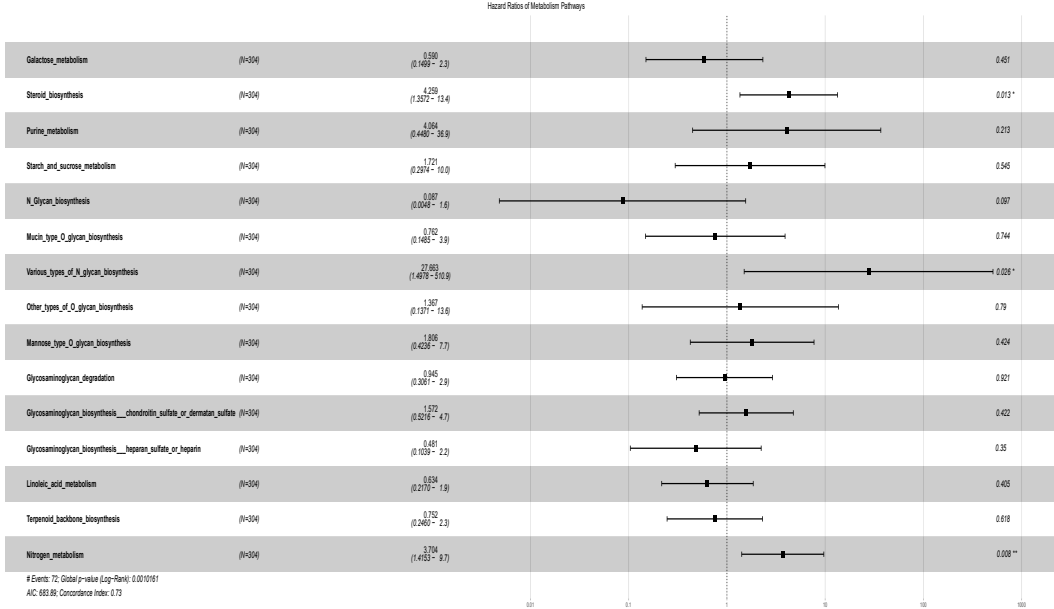

F

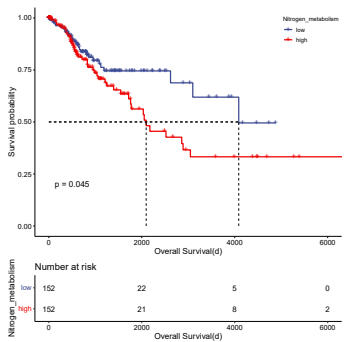

G

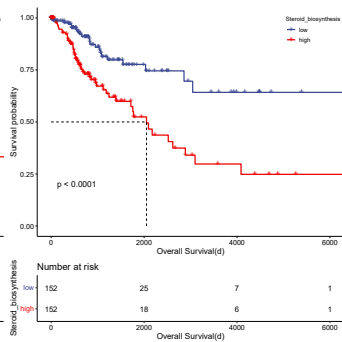

H

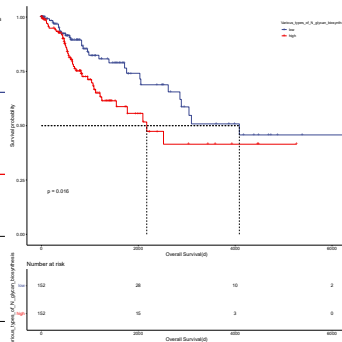

I

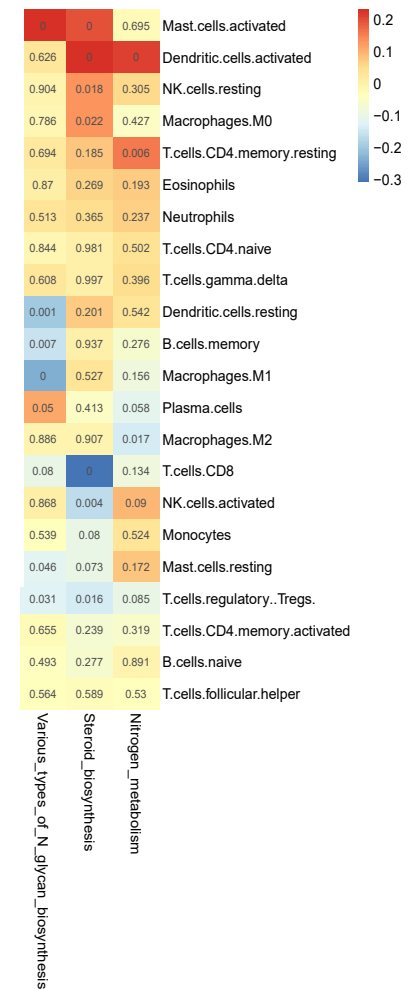

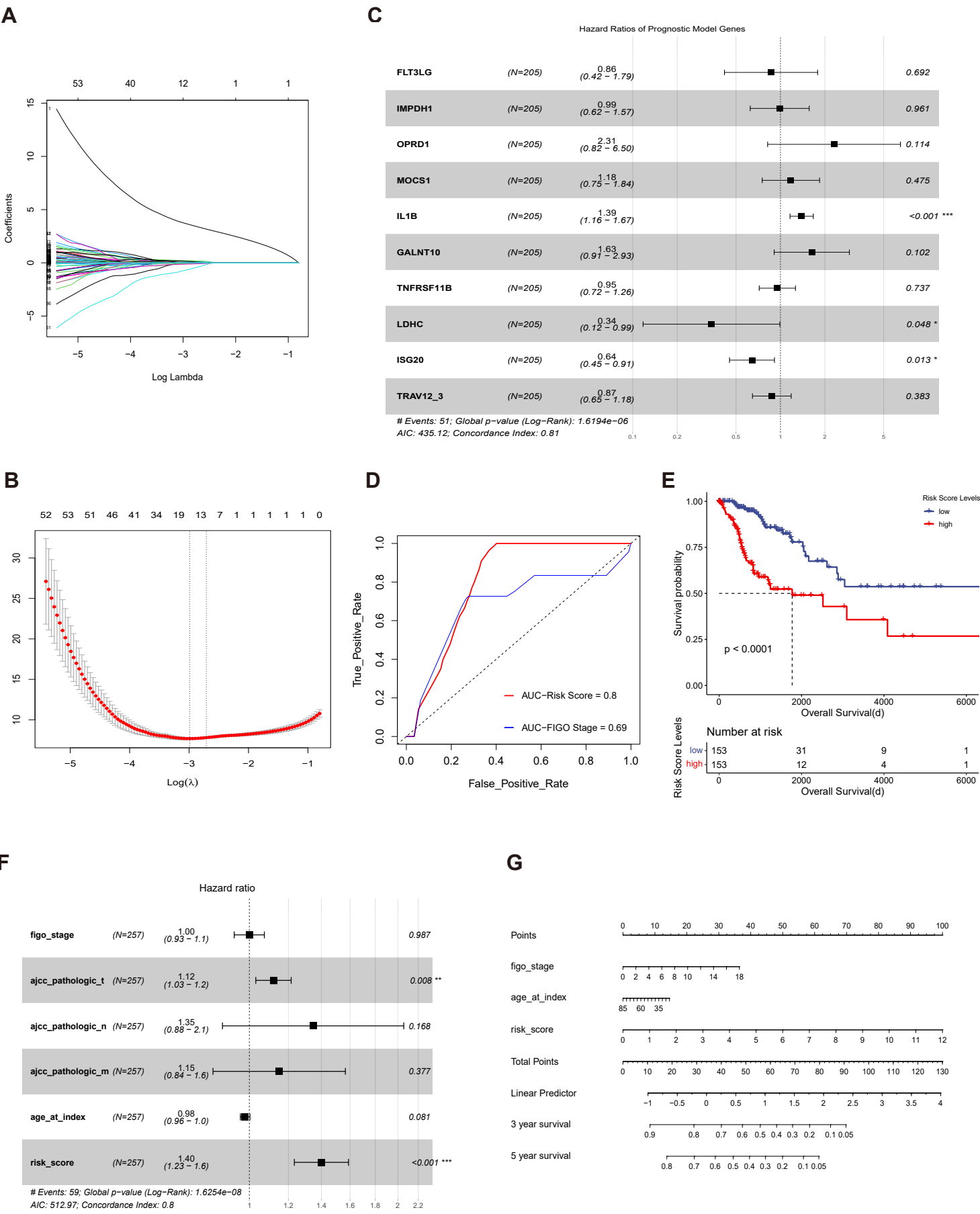

**A**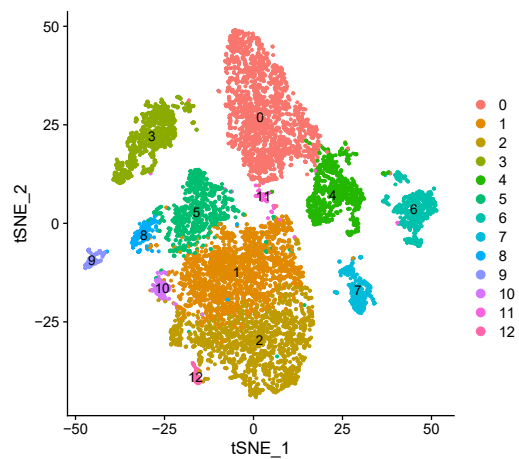**B**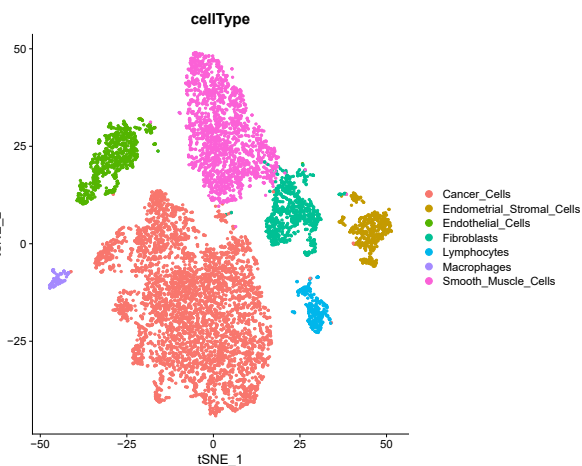**C**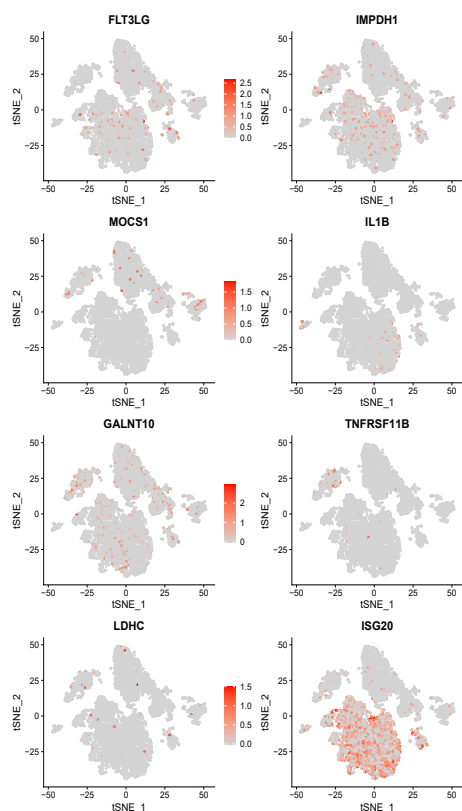**D**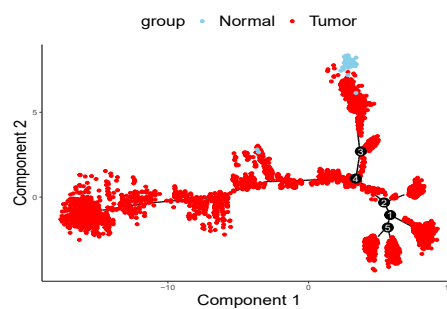**E**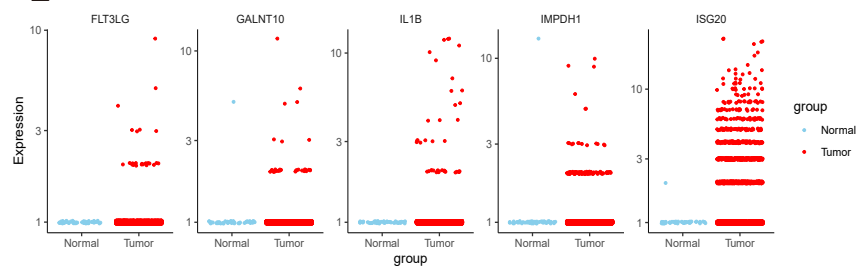**F**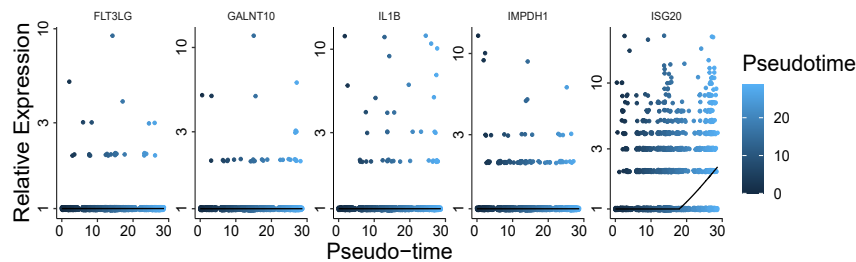**G**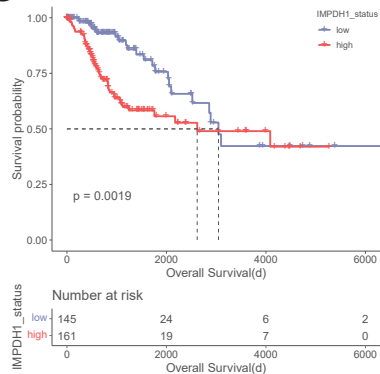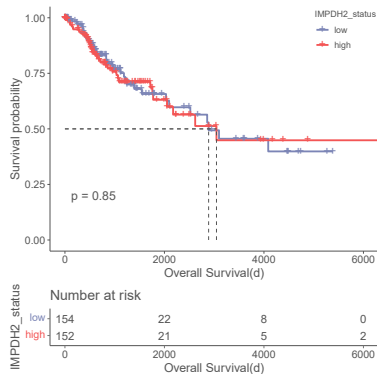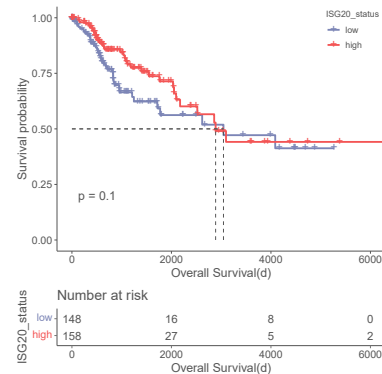

**A**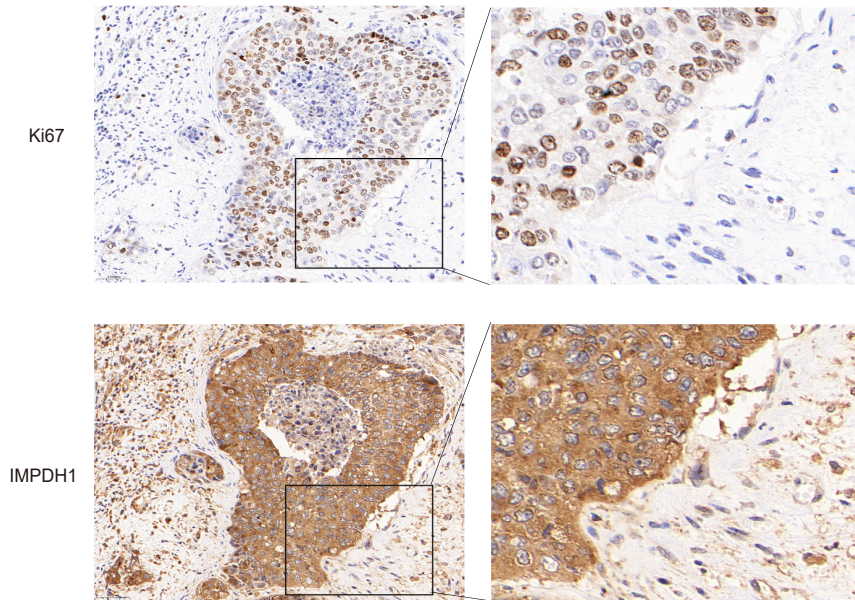**B**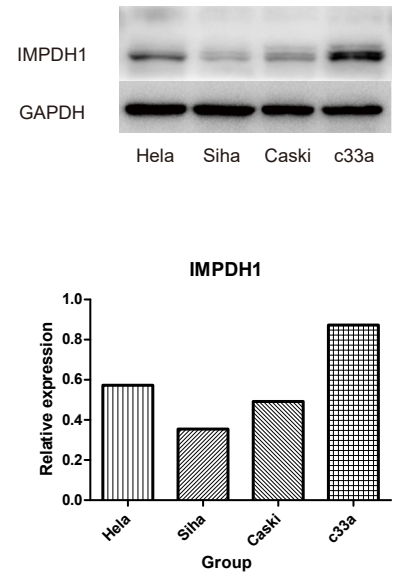**C**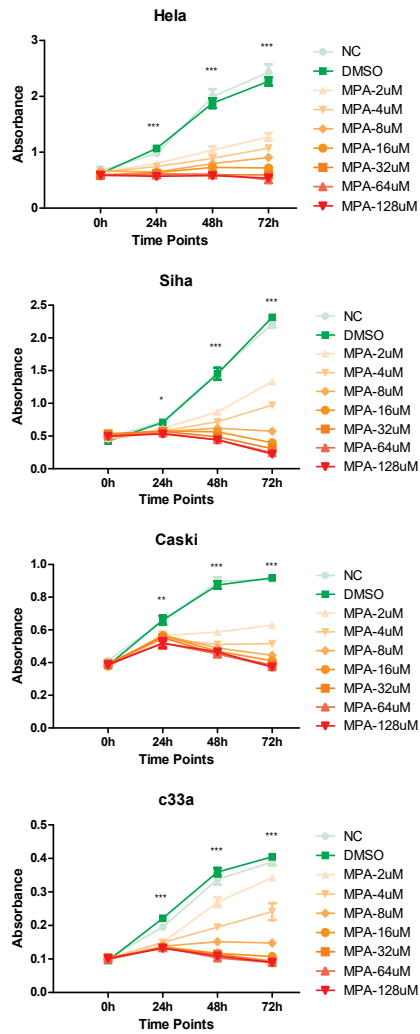**D**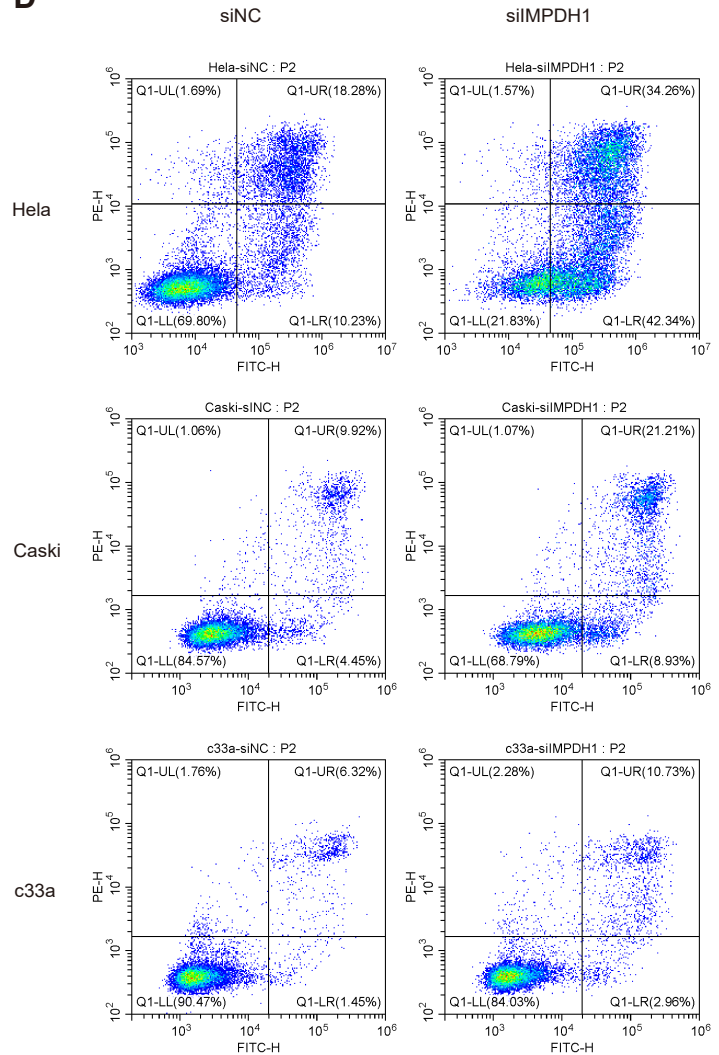

Supplement: Supplementary file 3 [file DataSheet1.PDF]
